# Regularization approaches in clinical biostatistics: A review of methods and their applications

**DOI:** 10.1177/09622802221133557

**Published:** 2022-11-16

**Authors:** Sarah Friedrich, Andreas Groll, Katja Ickstadt, Thomas Kneib, Markus Pauly, Jörg Rahnenführer, Tim Friede

**Affiliations:** 1 Institute of Mathematics, 26522 University of Augsburg, Augsburg, Germany; 2 Centre for Advanced Analytics and Predictive Sciences, University of Augsburg, Augsburg, Germany; 3 Department of Statistics, 14311 TU Dortmund University, Dortmund, Germany; 4 Chair of Statistics and Campus Institute Data Science, 84922 Georg-August-University Göttingen, Göttingen, Germany; 5 Department of Medical Statistics, University Medical Center Göttingen, Göttingen, Germany; 6 DZHK (German Center for Cardiovascular Research), partner site Göttingen, Göttingen, Germany

**Keywords:** Penalization, Bayesian inference, ensembling, model averaging, early stopping, evidence synthesis

## Abstract

A range of regularization approaches have been proposed in the data sciences to overcome overfitting, to exploit sparsity or to improve prediction. Using a broad definition of regularization, namely controlling model complexity by adding information in order to solve ill-posed problems or to prevent overfitting, we review a range of approaches within this framework including penalization, early stopping, ensembling and model averaging. Aspects of their practical implementation are discussed including available R-packages and examples are provided. To assess the extent to which these approaches are used in medicine, we conducted a review of three general medical journals. It revealed that regularization approaches are rarely applied in practical clinical applications, with the exception of random effects models. Hence, we suggest a more frequent use of regularization approaches in medical research. In situations where also other approaches work well, the only downside of the regularization approaches is increased complexity in the conduct of the analyses which can pose challenges in terms of computational resources and expertise on the side of the data analyst. In our view, both can and should be overcome by investments in appropriate computing facilities and educational resources.

## 1 Introduction

The general aim of regularization is to control model complexity by adding information, allowing us to solve ill-posed problems and prevent overfitting. With this broad definition, regularization includes techniques such as penalization,^1,2^ early stopping,^3,4^ ensembling^5,6^ and model averaging.^7^ These statistical techniques have been applied in medical research for some time now. For instance, penalization is implemented in variable or model selection through ridge regression^1^ or the least absolute shrinkage and selection operator (LASSO).^2^ These approaches can also be applied, in the context of missing data^8^ or causal analyses,^9^ to name a few. Furthermore, Bayesian hierarchical models are used for evidence synthesis.^10^ Whereas traditional meta-analysis focuses on the combined effect across a number of included studies, the same hierarchical models can also be utilized for dynamic borrowing, i.e. estimation of an effect in one study by borrowing information from the other studies, through shrinkage estimation.^11^ Clinical applications of regularization range from pharmacovigilance^12^ through non-small-cell lung cancer^13^ to Alzheimer’s disease.^14^

The concept of regularization has a long history in both mathematics and statistics. Many of the early approaches are well understood by now. In terms of adding information, the most prominent origin is Bayes’ idea of adding prior information to a likelihood based inference.^15,16^ The Bayesian model formulation with its prior and likelihood components per se allows for regularization. The grade of regularization then depends on the informativeness of the prior. While non-informative prior choices do not lead to regularization, vague, weakly informative and informative prior choices impose different levels of regularization. Tikhonov^17^ firstly aims to use regularization to solve an ill-posed problem. From a statistical perspective, Hoerl^18^ provides a ridge regression formulation of Tikhonov’s idea, and Foster^19^ interprets this method as a Wiener Kolmogoroff or Kriging filter. Tikhonov’s regularized solution can also be interpreted as a Bayes solution, see, e.g., Vogel^20^ or Wolpert and Ickstadt.^21^ Formally, adding information, e.g., in terms of a prior distribution, to a statistical inference problem is best described in a decision theoretic framework; see, e.g., Wald^22^ or Lehmann^23^ for the foundation of decision theory and Berger^24^ for a detailed overview. One of the first regularization ideas to avoid overfitting in a statistical analysis is the stepwise procedure of early stopping. Its origin lies in the theory of sequential testing and goes back to Wald.^25^ Nowadays, early stopping is employed in many statistical learning approaches.

Although there is a growing literature on regularization with a wealth of techniques being available to overcome the problems outlined above, it is currently largely unknown to what extent these methods are actually used in clinical medicine and what type of problems are addressed by their use. To shed some light on these questions we systematically reviewed recent volumes of three journals publishing in general medicine, namely the Journal of the American Medical Association (JAMA), the New England Journal of Medicine (NEJM) and the British Medical Journal (BMJ).

The remainder of this paper is organized as follows. In Section 2, an overview of regularization approaches is provided, starting with a brief history of regularization and in particular, covering aspects such as penalization, early stopping, ensembling and model averaging. In Section 3, a review of articles in medical journals that summarizes the current state of applications of regularization in clinical medicine is reported. Some examples are presented in Section 4 before making some closing remarks in Section 5.

## 2 Regularization approaches

In this section, we will describe a variety of regularization approaches. In particular, we will formulate specific goals as well as suitable statistical models and procedures to achieve them. The types of regularization approaches comprise penalization and including external and/or historical data (Section 2.1), early stopping (Section 2.2), ensembling (Section 2.3), and further ideas like injecting noise (Section 2.4). Table 1 summarizes all of these regularization types, their goals and the corresponding statistical methods. This section concludes with some practical remarks on regularization (Section 2.6) and corresponding software (Section 2.7).

**Table 1.** Overview of regularization types, their general idea, and the statistical approaches that fall into the respective category. The approaches are described in more detail in Sections 2.1 – 2.4.

Type	Description	Common statistical approaches
Penalization (Section 2.1)	Add penalty term(s) to fitting criterion	– Ridge regression, LASSO, elastic net – Bayesian regularization priors – Constraints for parameters – Random effects – Semiparametric regression
Early stopping (Section 2.2)	Early stopping of an iterative fitting procedure	– Coefficient paths in penalization approaches – Boosting – Pruning of trees – Learning rate in deep neural networks
Ensembling (Section 2.3)	Combine multiple base-procedures to an ensemble	– Bagging – Random forests – (Bayesian) model averaging – Boosting
Other approaches (Section 2.4)	–	– Injecting noise – Random probing in model selection – Out-of-sample evaluation

LASSO: least absolute shrinkage and selection operator.

### 2.1 Penalization

Penalization approaches make the trade-off between model fit and model complexity explicit by combining (a) a (lack of) fit criterion representing the ability of a model to fit the given data with (b) a penalty that measures the model complexity. In the following, we will introduce this idea in more detail for parametric models characterized by a parameter vector θ, but the ideas immediately generalize to semi- and non-parametric models. The observed data will be denoted as y and we will illustrate penalization along regression-type models where y represents a vector of observed response values while θ comprises the regression coefficients.

In a frequentist and loss-based formulation, penalized regularization approaches take the form

(1) ρ(y;θ)+pen(θ),

with some appropriately chosen loss function ρ(⋅;⋅) and non-negative penalty term pen(⋅). The loss function can be the negative log-likelihood of, e.g., a generalized linear model (GLM), but other loss functions such as L1 or L2 loss or robust versions such as Huber’s loss^26^ are also conceivable. The penalty term is chosen to reflect the complexity of the model (as characterized by the parameter vector θ) or to enforce desirable properties of the estimate. Popular examples include:

- The L2 penalty leading to ridge regression, where $\;\mathrm{pen}\;(\theta )=\lambda \theta^{⊤}\theta =\lambda \sum_{j}\theta_{j}^{2}$ with non-negative penalty parameter λ≥0, which enforces shrinkage and adds stability to the estimation.^1^
- The L1 penalty for simultaneous shrinkage and selection leading to the least absolute shrinkage and selection operator (LASSO) with $\;\mathrm{pen}\;(\theta )=\lambda \sum_{j}|\theta_{j}|$.^2,27–29^ Again the penalty enforces shrinkage and adds stability, but it also enables variable selection.
- The L0 penalty $\;\mathrm{pen}\;(\theta )=\lambda \sum_{j}1(\theta_{j}\neq 0)$ with the indicator function 1(⋅) implying a penalty on the number of non-zero coefficients.^29^

**Figure 1.**
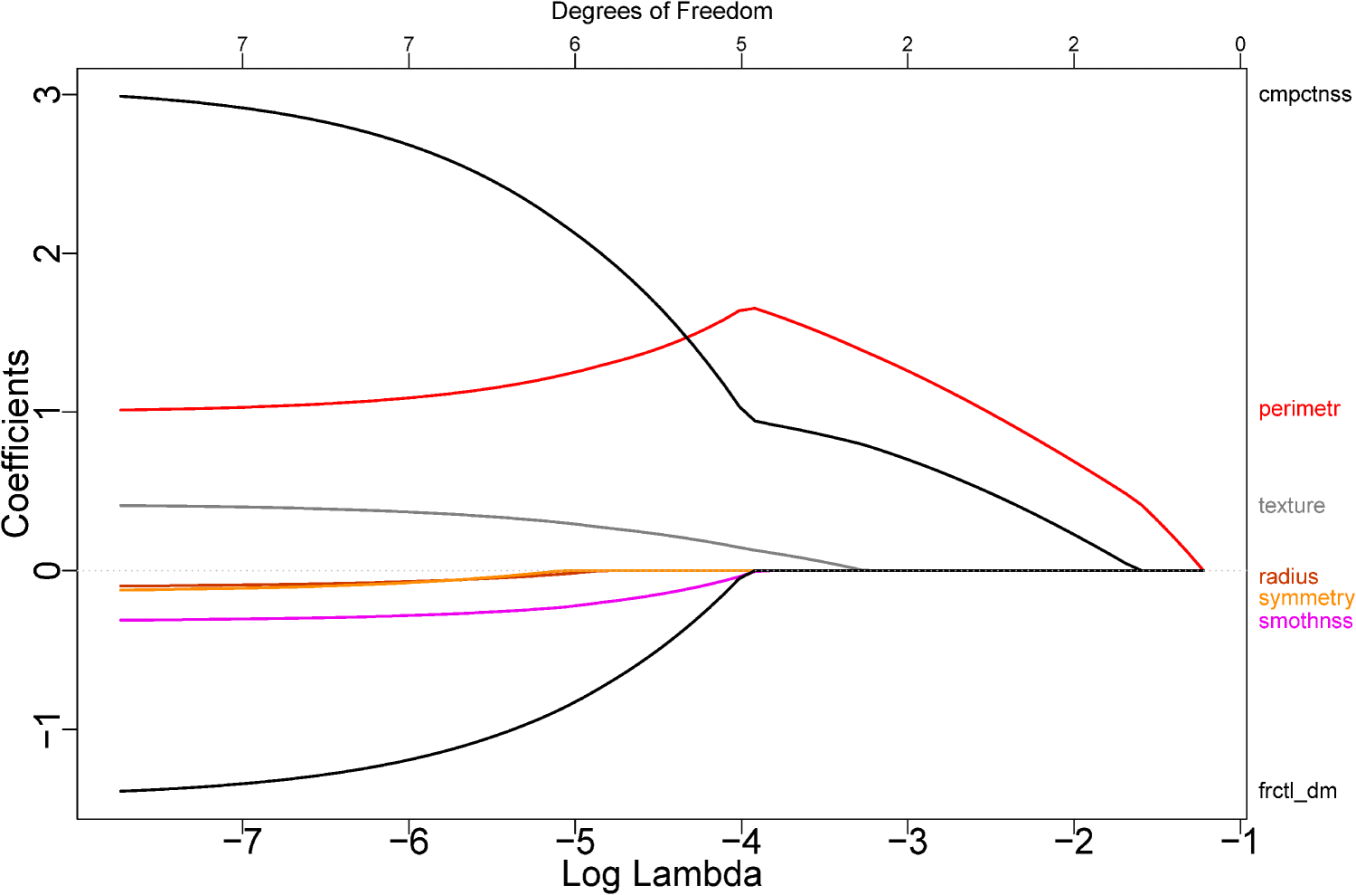
Coefficient paths obtained by applying the least absolute shrinkage and selection operator (LASSO) to the prostate data set discussed in the Section 2.7. As the penalty strength λ grows, the coefficients are shrunk towards zero.

In all these examples, the penalty includes a penalty parameter λ≥0 that governs the effective trade-off between the loss and the penalty term in (1). If λ→0, the penalty loses its importance such that the resulting estimate $\hat{\theta }$ minimizes the underlying loss irrespective of the chosen penalty (leading, e.g., to the maximum likelihood estimate in case of a loss function representing the negative log-likelihood), while for λ>0 the estimate minimizes the loss subject to a constraint imposed by the penalty. In the examples above, all penalties lead to an empty model with all parameters being estimated equal to zero, when λ approaches infinity. However, the paths at which the coefficients approach zero are very distinct and depend on the underlying geometry of the penalty term. In the case of orthonormal designs, ridge regression induces a proportional shrinkage of all coefficients, and therefore $\hat{\theta }=0$ is only achieved as a limiting case. For the LASSO, an orthonormal design, in contrast, leads to a linear decay to zero such that coefficients are exactly set to zero already for finite values of the penalty parameter λ. Figure 1 represents the coefficient paths for the LASSO applied to the prostate data set discussed in Section 2.7. Displayed are the paths for growing log(λ), illustrating the shrinking towards zero, which results in variable selection. One peculiarity here is that, due to the non-orthogonal design of the covariates, increasing the smoothing parameter may initially lead to increasing effect sizes. Still, when the smoothing parameter is increased further, all estimates eventually approach the limiting value of zero.

Various extensions and alternatives to the three penalties introduced above have been suggested to achieve other forms of penalization or to enforce other forms of the coefficients paths. For example, the penalty may leave certain parameter configurations unpenalized, such that even for λ→∞ there will be free parameters to estimate. As the simplest case, a number of parameters (such as the intercept and parameters relating to covariates that are deemed important a priori) may be left out of the penalization term. More complex models may also comprise multiple penalty parameters, for example when additively combining penalties such as in the elastic net^30^ with $\;\mathrm{pen}\;(\theta )=\lambda_{1}\sum_{j}\theta_{j}^{2}+\lambda_{2}\sum_{j}|\theta_{j}|$.

Penalized forms of regularized estimation enjoy a close link to Bayesian inference where, according to Bayes’ theorem, the posterior p(θ|y) can be determined as

$$p(\theta |y)=\frac{\;p(y|\theta )p(\theta )}{\int p(y|\theta )p(\theta )d\theta }\propto p(y|\theta )p(\theta )\;,$$

i.e. the posterior is proportional to the likelihood p(y|θ) times the prior p(θ).^2^ Taking the logarithm illuminates that (using ∝ to denote equality up to additive constants)

(2) log(p(θ|y))∝log(p(y|θ))+log(p(θ)),

such that maximizing the posterior is equivalent to a penalized estimate combining the log-likelihood with a penalty induced by the log-prior. This can be interpreted in two ways: On the one hand, regularized maximum likelihood estimates can also be understood as posterior mode estimates. On the other hand, the prior distribution in Bayesian inference determines a corresponding form of regularization with the log-prior inducing the penalty term. As such, regularization may also be interpreted as a way of including prior or expert knowledge in model estimation. Concerning the examples introduced above, ridge regression corresponds to an i.i.d. zero-mean Gaussian prior for the regression coefficients, while the LASSO has its equivalent in i.i.d. zero mean Laplace priors.^31^

In between frequentist, loss-based regularization and Bayesian regularization are models with random effects where some of the regression coefficients are assigned a random effects distribution that can formally also be interpreted as a Bayesian prior. Similarly, random effects estimates are often interpreted as shrinkage estimates where the random effects distribution enables estimation of a potentially large number of effects, shrunken towards zero. As a consequence, various types of models involving random effects, e.g., hierarchical mixed-effects models or spatial regression models involving spatially correlated stochastic processes, can also be seen as regularized regression where the specific form of regularization depends on the distributional assumption for the random effects. For example, most spatial regression models implement spatial dependence such that spatial effects tend to be similar when the corresponding locations are close to each other (corresponding to Tobler’s famous first law of geography stating that ‘everything is related to everything else, but near things are more related than distant things’^32^). In this case, the penalty implied by the distribution of the stochastic process penalizes large differences between spatial effects at close locations.

Finally, applying the penalty not directly to the parameter vector but to functions thereof allows to enforce other types of regularization behaviour. Furthermore, considering the penalty not on the original covariates but on transformations or basis function expansions thereof contributes further flexibility. Some areas that have attracted particular interest in the last decade include:

- *Fusion penalties*, where the goal is to fuse certain effects together, for example when considering the effects of features that can be ordered in some meaningful way. Effects of ordinal categorical covariates are just one particular example of this. One of the early suggestions is the ‘fused LASSO’^33^ that penalizes the L1 norm of both the coefficients and their successive differences, but several extensions have been suggested in the literature since then.^34–39^ A nice and extensive overview on the topic ‘Regularized regression for categorical data’, for both categorical predictors and responses, can be found in Tutz and Gertheiss.^40^ Also in the Bayesian framework, fusion of effects has been investigated by several researchers, see, e.g., Pauger et al.^41^ or Malsiner-Walli et al.^42^ A nice discussion on Bayesian regularization and effect smoothing for categorical predictors can be found in Wagner and Pauger.^43^
- *Semiparametric function estimation* with smoothness priors where a flexible effect f(x) of a covariate x of interest shall be estimated. One option is to work with function spaces and associated norms such as the functional L2 loss $\;\mathrm{pen}\;(\;\;f)=\lambda \int (\;\;f^{\prime \prime }(x){)}^{2}dx$, i.e., the integrated squared second derivative that penalizes the curvature of the function. This is the basis for the famous special case of smoothing splines. When approximating the effect of interest in terms of a basis expansion such that $f(x)=\sum_{j}\beta_{j}B_{j}(x)$ with appropriate basis functions $B_{j}(x)$, penalties can again be constructed for the basis coefficients $\beta_{j}$ with penalized splines^44,45^ as one of the most prominent examples. One can then also design penalties that enforce not only smoothness but other properties such as monotonicity, convexity/concavity or constant limiting behaviour.^46,47^
- *Structured additive regression models* that consider regression predictors
  $$f_{1}(\nu_{1})+\ldots +f_{j}(\nu_{j})+\ldots +f_{J}(\nu_{J})$$
  that are an additive combination of various types of effects $f_{j}(\nu_{j})$ based on covariate vectors of different type and associated with quadratic penalties to enforce desirable properties of the individual effects. For example, structured additive regression comprises nonlinear effects of continuous covariates, varying coefficient terms, interaction surfaces, random effects and spatial effects as special cases, see for example Fahrmeir et al.^48^ and Fahrmeir and Kneib^49^ for an in-depth discussion.
- *Single index models* that extend generalized linear and additive models by also estimating the link function that maps the regression predictor to the conditional expectation of the response variable in a data-driven way. When a flexible, nonparametric approach is taken for the link function, regularization is also required for this part of the model.^50^ With a linear predictor, single index models provide a combination of nonlinear and linear modelling techniques.

**Figure 2.**
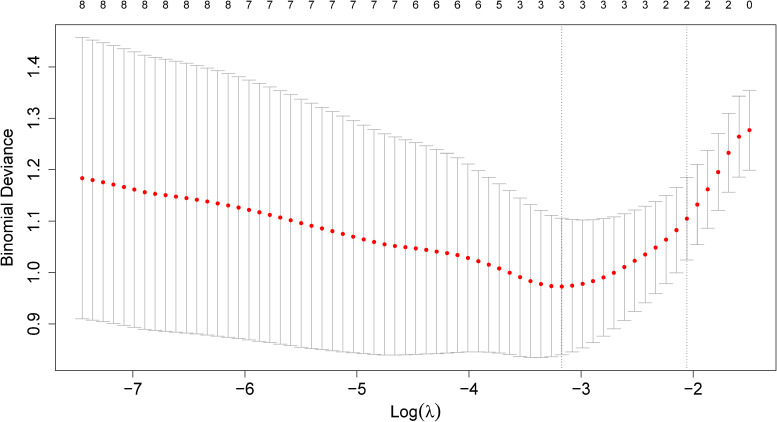
Cross-validation error curve for the LASSO applied to the prostate cancer data from Section 2.7. Two special values of λ are highlighted through vertical dotted lines: $\lambda_{\text{min}}$ gives the smallest mean cross-validated error (left), while $\lambda_{\text{1se}}$ is the value of λ that gives the most regularized model such that the cross-validated error is within one standard error of the minimum (right). The numbers on top of the plot denote the number of non-zero coefficients entering the model at the respective penalty strength.

We close the discussion of penalization approaches by highlighting that the penalty pen(⋅) from (1) typically involves one or multiple hyperparameters that determine the impact of the penalty on the fit. Determining the optimal hyperparameter(s) from the data allows for a data-driven amount of regularization and is a central problem for turning penalty-based regularization into practice. Cross-validation (CV) is one prominent example, but for specific classes of models, more specific approaches such as (restricted) maximum likelihood for determining random effects variances or smoothing parameters in structured additive regression are also conceivable, see for example Chs. 7 and 9 in Fahrmeir et al.^51^ Figure 2 shows the CV curves for the LASSO applied to the prostate cancer data, see Section 2.7 for details. In a Bayesian approach, suitable hyperpriors can be assigned to the hyperparameters, making them part of the Bayesian inferential scheme.

In R,^52^ penalization approaches such as LASSO or ridge regression are implemented in the packages glmnet^53^ and penalized.^54^

### 2.2 Early stopping

Many statistical and machine learning approaches build up a (potentially complex) model by iteratively refining a simple model towards the most complex case allowed by the model specification. One way of inducing regularization in such cases is to stop the fitting process before the most complex model is achieved, i.e. to identify the best trade-off between model simplicity (models close to the initial model) and fit to the data (models close to the final, most complex model) by early stopping. In fact, the penalization approaches discussed in the previous section can also be cast into this framework when considering the complete path of coefficients produced by varying the penalty parameter from infinity (simplest model determined by minimizing the penalty) to zero (complex model fit without the penalty). Early stopping then means that we are not using the most complex model without a penalty, but ‘stop’ at an optimal value for the penalty parameter determined, for example, again via CV techniques.

*Boosting approaches* are another example where regularization can be achieved by early stopping. The concept of boosting emerged from the field of machine learning^55^ and was later adapted to estimate predictors for statistical models.^56,57^ Main advantages of statistical boosting algorithms are their flexibility for high-dimensional data and their ability to incorporate variable selection in the fitting process.^3^ Furthermore, due to the modular nature of these algorithms, they are relatively easy to extend to new regression settings.^58^ In general, boosting algorithms can be also described as gradient descent approaches in function space,^4^ where the algorithm iteratively fits simple (e.g. linear) regression models (so-called base-learning procedures), not to the actual observations but to the negative gradient (first derivative) of the loss function – evaluated at the previous iteration. In this way, boosting iteratively improves the fit of the model by re-directing attention to those observations that have not yet been explained well and therefore still have large gradients. For a large number of iterations, the model will finally approach the minimizer of the loss function employed in the model specification. As a consequence, the number of boosting iterations is the main (and typically only) tuning parameter, and early stopping yields a regularized estimate determined by the starting values of the algorithm and the base-learners employed to generate the way towards the most complex model. In R, boosting is e.g. implemented in the packages mboost,^59^ gbm^60^ and xgboost.^61^

*Pruning of classification and regression trees* (CARTs) also fits into the range of early stopping procedures. Trees iteratively split the available data into subsets that are homogeneous with respect to some impurity measure within the subsets but maximize heterogeneity between the subsets. Taking this to the extreme, each observation would finally form its own subset, but usual tree implementations require a certain minimal number of observations in the final subsets, a strategy which already provides some (limited) protection against overfitting. However, the resulting trees are usually still too complex such that an additional pruning step is applied to remove superfluous splits.^62^ Consider the algorithmic generation of the full tree: This is an iterative procedure starting from a single set comprising all observations. From there, it moves over a simple stump with just two splits and finally becomes a fine-grained tree with many subsets. Early stopping now means that we determine the optimal number of splits based on some measure for generalizability such as CV. Software packages implementing these procedures are discussed in the context of random forests below.

As a final example, consider the *learning rate in deep neural networks*. Deep neural networks are usually trained with stochastic gradient descent optimization that updates the weights in the network. Due to a large number of weights involved in deep networks and the corresponding model flexibility, full optimization of the model would usually lead to a perfect fit, implying over-fitting and low generalizability. As a consequence, a decaying learning rate is usually implemented such that the maximum possible change is reduced as the model fit progresses. In effect, this means that after a certain number of iterations (dictated by the exact implementation of the decay of the learning rate), there will be no change in the weights of the network anymore, which also implements early stopping. Often an exponential decay is employed such that a single scalar value determines the learning rate and this parameter has to be chosen to achieve an optimal compromise between long-training processes (small learning rate) and unstable / over-fitting results (large learning rate). See Goodfellow et al.^63^ for details. Neural networks are implemented, among others, in the R-packages neuralnet^64^ and deepnet.^65^ Moreover, there exist interfaces to Python deep learning implementations such as keras.^66^

### 2.3 Ensembling and model averaging

While the previous two approaches build regularization directly into a specific model, we now turn to regularization by combining a variety of models with the aim of achieving improved model performance. For the sake of illustration, consider a model with a good ability to fit the given data but large variability such that the model does not generalize well to new data. If multiple variants of such a model are available, the variability can be reduced by building an ensemble of the models or by averaging over predictions or other quantities derived from the models.

We illustrate the idea of ensembling with one of the most frequently used ensemble techniques: *random forest*, originally proposed by Breiman.^6^ A random forest is an aggregation of a (typically large) number of classification or regression trees (which we already considered in the previous section). CARTs repeatedly partition the predictor space using binary splits of the covariate domain. The goal of the partitioning process is to find partitions such that the respective response values are very homogeneous within a partition but very heterogeneous between partitions (measured via criteria such as the mean squared error of prediction or the classification rate). CARTs can principally be used both for metric (regression trees) and for nominal/ordinal responses (classification trees). To obtain the prediction for a new observation, all response values within a partition are aggregated either by averaging (in regression trees) or simply by counting and using majority vote (in classification trees).

In the previous section, we already discussed pruning trees to avoid overfitting arising from trees with a large number of splits. However, even such pruned regression trees usually suffer from large estimation uncertainty, i.e. large variance, since small changes in the data can induce large differences in the resulting tree. To overcome this, random forests aggregate a large number B (e.g., B=5000) of trees grown independently from each other. The combination of many trees has the advantage that the resulting predictions inherit the property of unbiasedness from the single trees while reducing the variance of the predictions. To get a final prediction, predictions of single trees are aggregated (i.e. we form an *ensemble of trees*), in the case of regression trees simply by averaging over all the predictions from the single trees. In order to achieve the goal that the aggregation of trees is less variant than a single tree, it is important to reduce the dependencies between the trees that are aggregated in a forest. Typically, two randomization steps are applied to achieve this goal. First, the trees are not applied to the original sample but to bootstrap samples or random subsamples of the data. Second, at each node, a (random) subset of the predictor variables is drawn which is used to find the best split. These randomization steps de-correlate the single trees and help to lower the variance of a random forest compared to single trees. The size of the random subset of predictors at each node is a tuning parameter, which could be choosen e.g. by CV. According to Probst and Boulesteix,^67^ the number of trees B does not have to be tuned as long as it is chosen sufficiently large.

In R, two slightly different variants of regression forests are available. First, the classical random forest algorithm proposed by Breiman^6^ is implemented in the R-package ranger.^68^ The second variant is implemented in the function cforest from the party package.^69^ Here, the single trees are constructed following the principle of conditional inference trees as proposed in Hothorn et al.^70^ The main advantage of these conditional inference trees is that they avoid selection bias in cases where the covariates have different scales, e.g., numerical vs. categorical with many categories (see, for example, Strobl et al.^71,72^ for details). Conditional forests share the feature of conditional inference trees avoiding biased variable selection. The CV of the tuning parameter mtry can be done using the machine learning framework provided by the R-package mlr3.^73^

*Model averaging* is a second way of combining models together with the aim of improving upon the individual model performance. For illustration, let us consider a regression scenario where, from a set of p available covariates, we build all potential $2^{\;p}$ models. Instead of choosing one best model, e.g. based on some model choice criterion, we aim at combining the evidence for all models together, for example for forming predicted values. However, naively averaging over all models neglects differences in the ability of these models to explain the data as well as potential dependencies between the covariates. To overcome this, we weight the models according to some model fit criterion, for example the AIC. In this way, models that do not fit the data well obtain small weights and, vice versa, models with a good fit obtain large weights. If there is one single model that fits much better than all the others, the results from model averaging will be close to those from this model. However, in most cases, it is much more likely that there are multiple models with a similar fit that maybe only differ in small details. In such cases, all these models will contribute to the model-averaged prediction.

Model averaging can not only be used for forming predictions, but also for statistical inference on other properties shared by all models such as the regression coefficients or the variance. Intuitively, averaging over models again reduces the variance or the uncertainty associated with a single model. A thorough introduction to model averaging can be found in Claeskens and Hjort.^7^ The R-packages BMA^74^ and BMS^75^ implement Bayesian model averaging, whereas the model.avg-function of the MuMIn-package^76^ performs model averaging based on information criteria.

Other approaches that fit into the realm of model ensembling and model averaging include bagging^5^ and boosting (as discussed in the previous section), which iteratively combine weak learners. In general, we can distinguish between parallel modes of aggregation (where various models are fitted independently of each other and then combined together) and sequential ensembles (where the models are iteratively improved and then combined). Boosting is an example of the latter while random forests and model averaging are examples of the former.

### 2.4 Other regularization approaches

Of course, the list of regularization approaches discussed so far is by no means exhaustive. Various other takes on regularization exist, focusing on different aspects of the model-fitting process. One example is *injecting noise*, where some kind of distortion is introduced in the model fitting process. Random forests with their two steps of randomization (bootstrapping observations and considering only random subsets of covariates for splits) can be cast into this class as well and, hence, are a prominent example. In other cases, random probing, i.e. the introduction of simulated additional covariates that are, by definition, independent of the response of interest, can be used to better distinguish informative and non-informative covariates in model selection procedures such as boosting.^77^ Out-of-sample evaluation strategies can also be considered as an implicit mode of regularization, where the ability of the model to generalize well beyond the observed data is explicitly determined based on hold-out datasets. A further example is drop-out in neural networks, where part of the neurons in one layer is randomly shut down to avoid overfitting due to co-adaption.^78^

Many of the approaches discussed above are summarized in the R-package mlr3.^73^ In the supplementary material to this paper, we provide an example implementation of different regularization approaches using a data set on prostate cancer.^79^ Further details are discussed in Section 2.7 below.

### 2.5 Comparison of the different approaches

In this section, we briefly discuss pros and cons of the various regularization types and specific statistical approaches to provide guidance for applied users.

- *Flexibility.* While many of the statistical regularization approaches are tailored towards specific model classes and ways of modelling the data, penalization approaches (and partly also boosting approaches) are very flexible when it comes to implementing various types of constraints (e.g. complexity, sparsity, smoothness, etc.). This often implies advantages with respect to direct interpretability.
- *Interpretability.* While some methods are basically resulting in black box models, penalization and some of the early stopping methods (in particular, gradient- or likelihood-based boosting) can be used to estimate models that resemble unregularized versions when it comes to interpretation. This allows for an easier transition to practice. Nevertheless, some caution is still required since regularization results in biased estimates and often significance statements for those are not directly available anymore.
- *General loss functions.* Some of the methods, in particular penalization, boosting, random forests, and deep learning, allow to use general loss functions and not only least squares or log-likelihoods resulting from probabilistic models. This can have advantages to increase the robustness of the approaches.
- *Over-specified models.* Some methods are specifically tailored towards over-specified models, i.e. they allow to determine models with more covariates than observations or a large number of parameters resulting e.g. from basis function expansions or random effects. This includes penalization and Bayesian hierarchical approaches, but also boosting and deep learning.
- *Non-additive model specifications.* Sacrificing interpretation for the sake of better prediction, some approaches such as deep learning, bagging, and random forests allow for flexible covariate-response relations that circumvent the restrictions of additive model specifications.
- *Multi-model inference.* The ensembling approaches combine evidence from multiple models rather than focusing on one single ‘best’ model as most of the other approaches do.

### 2.6 Practical aspects with relevance to regularization

In any of the regularization approaches discussed before, there are a number of practical aspects that deserve particular attention when applying them in statistical analyses:

- *Interactions.* The inclusion of interaction effects in addition to main effects considerably increases the potential size of a statistical model. From this perspective, regularization is particularly interesting here since a large number of candidate effects (including interactions) can be generated, which is afterwards controlled via a suitable regularization approach. While most regularization approaches require the user to pre-define whether and which interactions shall be included, some automatically including potential interactions. For example, random forests implicitly implement interactions due to the recursive application of covariate splits.
  Note also that especially for regularization approaches enabling variable selection, e.g. the LASSO or componentwise boosting, particular care needs to be taken in order to account for certain hierarchical structures, such as ‘interaction effects only included if both main effects are included’.
- *Transformations of covariates.* Transforming covariates, for example, to account for specific types of nonlinear effects, is very common in regression analyses. Similar as with the interactions, transformations usually have to be pre-defined in regularized approaches. More precisely, regularization usually works on a pre-specified model class such as linear models, GLMs or generalized additive models (GAMs), and influences the specific version of the model estimated from the data but not the model class itself. Only when models are nested, as for example with the GLM and GAM, regularization may in fact reduce the more complex version to the simpler one.
- *Standardization.* Standardization is a specific type of transformation that can be useful or even necessary in regularization approaches. For example, penalization approaches such as the LASSO and ridge regression critically rely on the fact that all regression coefficients can be compared to each other in absolute terms. In such cases, standardizing all covariates is necessary to achieve this. Many software packages automatically perform the standardization step internally and report back-transformed estimates afterwards, but it is important to check the exact implementation to ensure that one interprets the estimated model correctly.
- *Covariate scales.* Similarly, covariates with different scales (e.g. continuous vs. categorical) can be problematic in regularization. For example, simple versions of random forests can be shown to have an intrinsic preference to select categorical covariates with more categories for the next split. While unbiased selection criteria have been suggested, the scale of covariates is still an important property to be considered in regularized approaches, in general.
- *Combining linear and nonlinear effects.* Principally, both for purely linear or nonlinear effects several approaches for variable selection via penalization exist. However, for the combination of both in so-called semiparametric models, suitable penalization is more tricky and only few works in this regard have been developed in the frequentist penalized likelihood framework.^80^ More work has been conducted based on the inherent model selection property of boosting^81–83^ and in the Bayesian framework based on variable and effect selection priors.^84,85^

### 2.7 R implementation of different regularization approaches

An example implementation of different regularization approaches can be found in the supplemental material to this paper. Here, we used a kaggle data set on prostate cancer for illustration purposes.^79^ The data contains information on the tumours of 100 patients (radius, texture, perimeter, etc.) as well as their diagnosis (binary outcome). We demonstrate the implementation of six different regularization approaches, namely a classification tree (CART), a random forest, subset selection, rigde regression, LASSO, and elastic net and compare them to standard logistic regression by means of area under the curve (AUC) and the mean classification error (MCE). Hyperparameters are chosen based on 10-fold CV and results are averaged over ten repetitions. In this example, standard logistic regression is outperformed by the regularization methods. The penalization approaches (LASSO, ridge and elastic net) perform better than logistic regression in terms of the AUC, while ridge regression, elastic net, CART, and subset selection have a smaller MCE than logistic regression.

## 3 The state of regularization applications in medicine

We performed a literature review in three top medical journals to investigate how much regularization is used in published medical research. To this aim, we reviewed all issues published between January and September 2020 in the Journal of the American Medical Association (JAMA) as well as in the New England Journal of Medicine (NEJM) and the British Medical Journal (BMJ). These journals were chosen since they range among the general medical journals with the highest impact factors in the world. We identified and reviewed all original research articles, resulting in 383 articles, see the PRISMA flow chart in the supplement.

### 3.1 Overview of used regularization methods

After the exclusion of three updates of a living systematic review, 380 articles remained. For each of these, the statistical methods section was screened for applications of regularization. Thereby, we used the definition and the examples of regularization approaches described in Section 2. The exact results of our search were collected in an Excel spreadsheet, which we provide as a supplement. It contains the following general information for each paper: the digital object identifier, the journal in which it was published (JAMA, NEJM or BMJ), the name of the first author, and the title. As statistically relevant variables it also includes the studies’ sample sizes, a dichotomous variable describing whether regularization was used and, if so, another one indicating the exact form of regularization as described in Section 2. Moreover, we extracted the type of software used for the analyses. Our main findings regarding the use of regularization are summarized in Table 2. In the supplement, we provide an additional table summarizing the study characteristics.

**Table 2.** Number of regularization applications found from our literature review. As some regularization methods were occasionally used in combination, multiple enumerations are possible. Row-wise percentages are rounded to integers.

	No regularization	Random effects	Bayes	Penalization	A priori	CV	Smoothing	Boosting	Random forest	Subset selection
JAMA	62 (61%)	35 (35%)	1 (1%)	2 (2%)	0 (0%)	1 (1%)	0 (0%)	1 (1%)	1 (1%)	0 (0%)
NEJM	121 (74%)	31 (19%)	8 (5%)	1 (1%)	2 (1%)	1 (1%)	2 (1%)	0 (0%)	0 (0%)	0 (0%)
BMJ	70 (60%)	38 (33%)	7 (6%)	3 (3%)	1 (1%)	0 (0%)	1 (1%)	1(1%)	0 (0%)	1 (1%)
Total	253 (67%)	104 (27%)	16 (4%)	6 (2%)	3 (1%)	2 (1%)	3 (1%)	2 (1%)	1 (0%)	1 (0%)

CV: cross-validation; JAMA: Journal of the American Medical Association; NEJM: New England Journal of Medicine; BMJ: British Medical Journal.

The two striking key messages are as follows:

1. The majority of studies did not use regularization techniques at all.
2. If regularization was used, it was mainly by means of random effects.

In fact, out of the 128 studies that applied at least one regularization method, 104 used random effect models. Other techniques that were used cover Bayesian ( 16 out of 380) and penalization methods ( 6) as well as smoothing ( 3), a priori knowledge ( 3), CV ( 2) and boosting ( 2). Random forests and subset selection were only used once, respectively. The numbers also suggest a different journal openness regarding regularization: While 39% (SE 0.048) of the JAMA articles and 40% (SE 0.045) of the BMJ articles applied regularization, only 26% (SE 0.034) of the NEJM articles did.

### 3.2 Discussion of specific examples

For each regularization method, other than random effects modelling, listed in Table 2 we briefly discuss its concrete usage in the reviewed papers In the remainder of this section, we refrain from citing these papers in the references, since they serve as examples rather than literature citations. Complete information can be found in the online supplement.

*Bayesian methods* were used in 16 papers as follows: In a randomized control trial (RCT) on coronary heart disease, Maron et al. (2020, NEJM) used Bayesian techniques to quantify effect sizes. In the context of coronavirus disease 2019 (COVID-19), Li et al. (2020, NEJM) used an informative prior distribution from SARS studies to construct a serial interval, while Reynolds et al. (2020, NEJM) used Bayesian methods to compare outcomes between treated and untreated patients. Hong et al. (2020, NEJM) used a Bayes-logistic regression model for dose-escalation in a lung cancer study. A Bayesian random effects model with informative priors for heterogeneity estimates was used by Ferreyro et al. (2020, JAMA) for a research synthesis on acute hypoxemic respiratory failure. Spertus et al. (2020, NEJM) used Bayesian methods in the context of two RCT studies (on kidney and coronary disease, respectively) with longitudinal data. Chen et al. (2020, NEJM) applied a Bayesian linear modelling framework for plasma proteome analyses in undernourished children. In the context of coronary heart disease, Bangalore et al. (2020, NEJM) used a Bayesian approach to assess the effect of revascularization on a composite endpoint. Bayesian meta-analyses were used by Siemieniuk et al. (2020, BMJ), Ge et al. (2020, BMJ), Wang et al. (2020, BMJ), Moustgaard et al. (2020, BMJ), Parisi et al. (2020, BMJ) and Li et al. (2020, BMJ) in different application contexts. Richardson et al. (2020, BMJ) used a Bayesian linear mixed model in a Mendelian randomization study.

Six papers used *penalization*: Kang et al. (2020, NEJM) used a penalized Cox model with Firth correction in an RCT on severe aortic stenosis. To tune polygenic risk scores in an observational study on coronary artery disease, Elliott et al. (2020, JAMA) applied Lassosum. Three papers used penalized splines: Knight et al. (2020, BMJ) in a study on COVID-19, Ho et al. (2020, BMJ) in a study on cardiovascular disease and Huang et al. (2020, BMJ) in a prospective cohort study on stroke. Dieleman et al. (2020, JAMA) used penalized regression to avoid spurious associations caused by small sample sizes and applied CV for the determination of the tuning parameters. CV was also applied by Milea et al. (2020, NEJM) for classification of fundus photographs.

*A priori information* in the sense of domain knowledge was used to impute missing values in two studies on bacterial infection (Jernigan et al., 2020, NEJM and Guh et al., 2020, NEJM), while Lewnard et al. (2020, BMJ) used a priori information borrowed from previous studies to develop parameterizations of the incubation period in COVID-19.

*Smoothing* was applied in three papers: Peled et al. (2020, NEJM) used loess-smoothed averages in plots for microbiota composition in patients undergoing haematopoietic-cell transplantation. In a study on rheumatoid arthritis, Orange et al. (2020, NEJM) applied the locally weighted scatterplot smoothing (LOWESS) technique. Safiri et al. (2020, BMJ) used smoothing spline models to determine the shape of the association between neck pain burden and sociodemographic indices.

*Boosting* was used twice, namely by Dhruva et al. (2020, JAMA), who applied extreme gradient boosting for the development of a log-odds model with high-dimensional nonlinear relationships between covariates, and by Knight et al. (2020, BMJ), who used gradient boosting decision trees in a study on COVID-19.

Finally, a *random survival forest* was used by Fosbol et al. (2020, JAMA) in a study on COVID-19 as a sensitivity analysis, and Nicholson et al. (2020, BMJ) performed *subset selection* in a logistic regression model for cancer.

On the other hand, several papers did not use regularization approaches although the analyses could have benefited from it. For example, instead of using GEEs, Pincus et al. (2020, JAMA), Lindenauer et al. (2020, JAMA), Marshall et al. (2020, NEJM), Juul et al. (2020, NEJM) and Marc et al. (2020, JAMA), among others, could have used random effect models. In Lindenauer et al., for instance, modelling the hospital clusters as random effects would have enabled a different kind of analysis, where the focus is on the conditional rather than the marginal effect.^86^ Other papers, e.g. Piccininni et al. (2020, BMJ) and Man et al. (2020, JAMA), used linear splines with fixed knots. Here, penalized splines might increase the flexibility of the model and help detecting nonlinear effects. Variable selection methods could have been applied in numerous papers to reduce the number of covariates included in the models. Examples include Kurth et al. (2020, JAMA), Pasternak et al. (2020, BMJ), Bailey et al. (2020, NEJM) or Smith et al. (2020, JAMA).

This concludes the overview of the use of regularization methods applied in real studies published in 383 research papers. It is our opinion that regularization would have been beneficial as well for several other studies, since they increase flexibility and can combine evidence from multiple models or sources, as mentioned in Section 2. We illustrate this in the next section, by explaining the concrete approach and the obtained results of three different regularization applications in the literature.

## 4 Examples

To demonstrate the versatility of using regularization methods and their potential positive effects, we discuss selected biostatistical examples from the literature.

### 4.1 Variable selection and shrinkage methods for linear regression

An example well-known in the statistical learning community refers to the prostate cancer data set analysed in Chapter 3.4 of Hastie et al.^87^ with shrinkage methods for linear regression. The data originate from a study by Stamey et al.^88^ who examined the level of prostate-specific antigen (PSA) in 97 prostate cancer patients, before receiving radical prostatectomy. PSA is a well-known biomarker in prostate cancer and the correlation of log PSA (lpsa) to eight clinical variables was analysed, including log cancer volume (lca), log prostate weight (lweight), age, log of benign prostatic hyperplasia amount (lbph), seminal vesicle invasion (svi), log of capsular penetration (lcp), Gleason score (gleason), and percent of Gleason scores 4 or 5 (pgg45).

A natural approach to model the relationship between lpsa and the eight predictors in a multivariate approach is standard linear regression, without any regularization. At first glance, in this case, variable selection seems not urgently required (only p=8 variables and n=97 observations). However, Hastie et al.^87^ applied various variable selection and shrinkage methods to the regression problem. First, the data were split into a training set and a test set. Then, on the training set, the models were fitted using CV for potential hyperparameter tuning. Finally, test set errors were computed on the test set that was not touched for model fitting, and its standard errors were estimated on the left-out sets in the CV.

In best subset selection, all combinations of variables were considered. Ridge regression and LASSO regression were used as shrinkage methods, and principal component regression and partial least squares as methods with decorrelated linear combinations of the original variables. The results are presented in Table 3, cf. Table 3.3 in Hastie et al.^87^

**Table 3.** Results for the application of different (mostly regularized) regression models to the prostate cancer dataset, with model fitting on the training set, using 10-fold CV. Reported are test errors, their estimated standard errors computed from the CV, and numbers of variables selected.

	Least squares	Best subset	Ridge regression	LASSO regression	Principal component regression	Partial least squares
Test error	0.521	0.492	0.492	0.479	0.449	0.528
Std error	0.179	0.143	0.165	0.164	0.105	0.152
Number of variables	8	2	8	4	8	8

CV: cross validation; LASSO: least absolute shrinkage and selection operator.

The test error was highest for standard linear regression (least squares) and for partial least squares and considerably lower for all other approaches. Further, best subset and LASSO regression selected only two and four variables out of the original eight, respectively. This demonstrates the potential benefit from regularization, here in the form of penalized regression, even in this fairly simple situation, due to the considerable correlation between the original variables.

### 4.2 Using additional a priori information for evidence synthesis

Borrowing information, for example from an observational study, to support a small-scale randomized trial can be achieved by deriving a shrinkage estimate within a Bayesian random effects meta-analysis.^11^ The approach first analyses the observational data using the shrinkage estimator in a hierarchical model and subsequently uses the derived posterior distribution to inform the analysis of the RCT. The efficiency gain of this approach was exemplified by Röver and Friede^11^ in the context of Creutzfeld-Jakob disease. This disease is a rare disease with a prevalence of 1 in 1,000,000. An RCT on doxycycline^89^ was terminated prematurely with only 12 patients included. However, additional data on 88 patients was available from an observational study. The primary endpoint of all-cause mortality was analysed using Cox proportional hazards regression. Using the external information from the observational study led to Bayesian shrinkage intervals spanning only two-thirds of the confidence interval derived from RCT data, thus showing a clear gain in efficiency.

The approach was also applied in a recent study^90^ on children with Alport syndrome. The Alport syndrome is a rare kidney disease which typically leads to end-stage renal disease in early life and requires renal replacement.^91^ Incorporating the results of real-world evidence from a prospective US cohort into the randomized data by Bayesian evidence synthesis resulted in a more precise estimate of the treatment effect indicated by a much shorter credible interval.

### 4.3 Boosting capture-recapture methods

Systematic reviews of clinical trials should be based on all relevant trials on the particular topic. For evaluation of the comprehensiveness of systematic literature reviews, capture-recapture analyses have been proposed. These require the selection of an appropriate model. To this end, Rücker et al.^92^ proposed to combine capture-recapture analysis with componentwise boosting. The boosting procedure allows to specify the mandatory variables that are always included in the model as well as optional variables. The latter are included only if relevant. This approach turned out to be robust against overfitting, and an appropriate model for statistical inference was automatically developed. In particular, Rücker et al.^92^ compared the componentwise boosting to a manually selected Poisson model to estimate the number of missing references for two systematic reviews on gastroenterology (prevention of biliary stent occlusion) and haematology (managing transfusional iron overload in sickle cell disease patients), respectively. For the first study, the manually selected model estimated 82 missing articles (95 % CI: 52–128), whereas the boosting technique found 127 (95 % CI: 86–186) missing articles. For the second example, boosting again provided a more efficient estimate of 188 (95 % CI: 159–223) compared to the best manually selected model (140 missing articles with 95 % CI: 116–168).

## 5 Discussion

A range of regularization approaches has been proposed to overcome problems such as overfitting, deal with data sparsity or improve the prediction and generalizability of results. Using a broad definition of regularization, namely the process of adding information in order to control model complexity, we reviewed a range of approaches within this framework including penalization, early stopping, ensembling and model averaging. We discussed aspects of their practical implementation including available R-packages. In this manuscript, we focused on R as a programming language and also demonstrated the use of regularization methods in an R-implementation. However, regularization approaches are also implemented in other statistical software. For example, penalization approaches such as LASSO or Ridge regression are implemented in SAS in the GLMSELECT and the REG procedure, while more complex penalization methods can be found in PROC TPSPLINE. A random forest implementation is given by PROC HPFOREST, for example. Bayesian method can be incorporated in various ways: PROC FMM, PROC GENMOD, PROC LIFEREG and PROC PHREG allow for Bayesian analyses through a BAYES statement, while PROC BGLIMM and PROC MCMC are specifically tailored to perform Bayesian estimation.^93^ Similarly, xtreg, lasso and boost provide implementations of random effects models, LASSO penalization and boosting in Stata, respectively. Examples were provided to showcase the practical use of regularization encouraging more wide spread use of these techniques in medicine. This is on the background of our review of recent issues of three general medical journals, which revealed that regularization approaches could be used more. The only exception are random effects models which featured relatively regularly. Other regularization approaches were rarely applied. In our view, there is space for improvement in the use of regularization methods in clinical medicine. Their application can be considered on a regular basis, since they only improve analyses and their interpretation. In situations where also other approaches work well, the only downside of the regularization approaches is increased complexity in the conduct of the analyses which can pose challenges in terms of computational resources and expertise on the side of the data analyst. In our view, both can and should be overcome by investments in appropriate computing facilities and educational resources.

Of course, the application of regularization approaches also entails some limitations:

- In general, their application is somewhat more challenging and requires a better understanding of the corresponding methodology. However, as we have shown in this review, the methodological gap between classical and regularized approaches is not always that big in the end.
- Some of the regularization approaches presented in this manuscript can be computationally intense, e.g. in the case of neural networks or random forests, especially when cross-validated tuning of hyperparameters is required.
- Some approaches are black box methods (e.g. tree-based ensembles, or deep neural networks), and will therefore be hard to interpret, limiting the possibility to communicate them to practitioners.

Finally, the selection of any statistical approach should of course be guided by the actual research question at hand and the corresponding goals (e.g. causal vs. exploratory vs. predictive analyses), and regularization approaches will not always be the best choice for this goal. For example, Riley et al.^94^ demonstrated that penalization and shrinkage methods produced unreliable clinical prediction models especially when the sample size was small.

The review of NEJM, JAMA and BMJ shed some light on the current state of the use of regularization methods in medicine. Although the review clearly shows that regularization methods are underused in clinical applications, it is limited in scope since only three journals were searched. Moreover, the choice of journals to include in such a review remains somewhat arbitrary. For instance, one reviewer suggested to include The Lancet as an additional high-impact general medicine journal. Furthermore, we focused on a relatively recent time period only and did not investigate any trends over time.

Before us, others have highlighted that existing methods are underused in clinical applications leading to suboptimal designs and analyses, sometimes even resulting in misleading interpretations. As an example, we refer to the STRATOS (STRengthening Analytical Thinking for Observational Studies) initiative (https://stratos-initiative.org/).^95^ Several topic groups (TGs) of the initiative are also concerned with regularization approaches, in particular, TG 2 ‘Selection of variables and functional forms in multivariable analysis’^96^ and TG 9 ‘High-dimensional data’.

## Supplemental Material

sj-pdf-1-smm-10.1177_09622802221133557 - Supplemental material for Regularization approaches in clinical biostatistics: A review of methods and their applications Click here for additional data file. Supplemental material, sj-pdf-1-smm-10.1177_09622802221133557 for Regularization approaches in clinical biostatistics: A review of methods and their applications by Sarah Friedrich, Andreas Groll, Katja Ickstadt, Thomas Kneib, Markus Pauly, Jörg Rahnenführer and Tim Friede in Statistical Methods in Medical Research

sj-csv-2-smm-10.1177_09622802221133557 - Supplemental material for Regularization approaches in clinical biostatistics: A review of methods and their applications Click here for additional data file. Supplemental material, sj-csv-2-smm-10.1177_09622802221133557 for Regularization approaches in clinical biostatistics: A review of methods and their applications by Sarah Friedrich, Andreas Groll, Katja Ickstadt, Thomas Kneib, Markus Pauly, Jörg Rahnenführer and Tim Friede in Statistical Methods in Medical Research

## References

[1] Hoerl AE Kennard RW. Ridge regression: biased estimation for nonorthogonal problems. Technometrics 1970; 12: 55–67.

[2] Tibshirani R. Regression shrinkage and selection via the lasso. J R Stat Soc Series B Stat Methodol 1996; 58: 267–288.

[3] Mayr A Binder H Gefeller O, et al. The evolution of boosting algorithms - from machine learning to statistical modelling. Methods Inf Med 2014; 53: 419–427. 25112367 10.3414/ME13-01-0122

[4] Bühlmann P Hothorn T. Boosting algorithms: regularization, prediction and model fitting (with discussion). Stat Sci 2007; 22: 477–505.

[5] Breiman L. Bagging predictors. Mach Learn 1996; 24: 123–140.

[6] Breiman L. Random forests. Mach Learn 2001; 45: 5–32.

[7] Claeskens G Hjort NL. Model selection and model averaging. Cambridge, UK: Cambridge University Press, 2008.

[8] Ch Tseng H Chen Y. Regularized approach for data missing not at random. Stat Methods Med Res 2019; 28: 134–150. 28671033 10.1177/0962280217717760 PMC7162734

[9] Ye Z Zhu Y Coffman DL. Variable selection for causal mediation analysis using LASSO-based methods. Stat Methods Med Res 2021; 30: 1413–1427. 33755518 10.1177/0962280221997505 PMC8189011

[10] Spiegelhalter DJ Abrams KR Myles JP. Bayesian approaches to clinical trials and health-care evaluation. vol. 13. Chichester: John Wiley & Sons, 2004.

[11] Röver C Friede T. Dynamically borrowing strength from another study through shrinkage estimation. Stat Methods Med Res 2020; 29: 293–308. 30821201 10.1177/0962280219833079

[12] Ahmed I Pariente A Tubert-Bitter P. Class-imbalanced subsampling lasso algorithm for discovering adverse drug reactions. Stat Methods Med Res 2018; 27: 785–797. 27114328 10.1177/0962280216643116

[13] Li Y Wang F Li R, et al. Semiparametric integrative interaction analysis for non-small-cell lung cancer. Stat Methods Med Res 2020; 29: 2865–2880. 32281490 10.1177/0962280220909969

[14] Li S Wu Q Sun J. Penalized estimation of semiparametric transformation models with interval-censored data and application to Alzheimer’s disease. Stat Methods Med Res 2020; 29: 2151–2166. 31718478 10.1177/0962280219884720

[15] Bayes F. An essay towards solving a problem in the doctrine of chances. by the late Rev. Mr. Bayes, F. R. S. communicated by Mr. Price, in a letter to John Canton, A. M. F. R. S. Philos Trans R Soc Lond 1763; 53: 370–418.

[16] Barnard GA Bayes T. Studies in the history of probability and statistics: Ix. Thomas Bayes’s essay towards solving a problem in the doctrine of chances. Biometrika 1958; 45: 293–315.

[17] Tikhonov AN. On the stability of inverse problems. In *Dokl. Akad. Nauk SSSR*, volume 39. pp. 195–198.

[18] Horel A. Applications of ridge analysis toregression problems. Chem Eng Prog 1962; 58: 54–59.

[19] Foster M. An application of the Wiener-Kolmogorov smoothing theory to matrix inversion. J Soc Indus Appl Math 1961; 9: 387–392.

[20] Vogel CR. Computational methods for inverse problems. Philadelphia: Society for Industrial and Applied Mathematics, 2002. ISBN 0-89871-550-4.

[21] Wolpert RL Ickstadt K. Reflecting uncertainty in inverse problems: a Bayesian solution using Lévy processes. Inverse Probl 2004; 20: 1759–1771.

[22] Wald A. Contributions to the theory of statistical estimation and testing hypotheses. Ann Math Stat 1939; 10: 299–326.

[23] Lehmann EL. Some principles of the theory of testing hypotheses. In *Selected Works of EL Lehmann*. Springer, 2012. pp. 139–164.

[24] Berger JO. Statistical decision theory and Bayesian analysis. New York: Springer Science & Business Media, 2013.

[25] Wald A. Sequential tests of statistical hypotheses. Ann Math Stat 1945; 16: 117–186.

[26] Huber PJ. Robust estimation of a location parameter. Ann Math Stat 1964; 35: 73–101.

[27] Tibshirani R. The lasso method for variable selection in the Cox model. Stat Med 1997; 16: 385–395. 9044528 10.1002/(sici)1097-0258(19970228)16:4<385::aid-sim380>3.0.co;2-3

[28] Friedman J Hastie T Tibshirani R. Regularization paths for generalized linear models via coordinate descent. J Stat Softw 2010; 33: 1. 20808728 PMC2929880

[29] Hastie T Tibshirani R Wainwright M. Statistical learning with sparsity – the lasso and generalizations. Boca Raton: Chapman and Hall/CRC, 2015.

[30] Zou H Hastie T. Regularization and variable selection via the elastic net. J R Stat Soc Ser B Stat Methodol 2005; 67: 301–320.

[31] Park T Casella G. The Bayesian lasso. J Am Stat Assoc 2008; 103: 681–686.

[32] Tobler W. A computer movie simulating urban growth in the Detroit region. Econ Geogr 1970; 46: 234–240.

[33] Tibshirani R Saunders M Rosset S, et al. Sparsity and smoothness via the fused lasso. J R Stat Soc Ser B Stat Methodol 2005; 67: 91–108.

[34] Gertheiss J Tutz G. Penalized regression with ordinal predictors. Int Stat Rev 2009; 77: 345–365.

[35] Gertheiss J Tutz G. Sparse modeling of categorial explanatory variables. Ann Appl Stat 2010; 4: 2150–2180.

[36] Schauberger G Groll A Tutz G. Analysis of the importance of on-field covariates in the German Bundesliga. J Appl Stat 2018; 45: 1561–1578.

[37] Groll A Hambuckers J Kneib T, et al. Lasso-type penalization in the framework of generalized additive models for location, scale and shape. Comput Stat Data Anal 2019; 140: 59–73.

[38] Shin S Fine J Liu Y. Adaptive estimation with partially overlapping models. Stat Sin 2016; 26: 235. 26917931 10.5705/ss.2014.233 PMC4762277

[39] Tang L Song PX. Fused lasso approach in regression coefficients clustering: learning parameter heterogeneity in data integration. J Mach Learn Res 2016; 17: 3915–3937. PMC5647925 29056876

[40] Tutz G Gertheiss J. Regularized regression for categorical data. Stat Modelling 2016; 16: 161–200. 10.1177/1471082x16645694 PMC5319706 28239293

[41] Pauger D Wagner H. Bayesian effect fusion for categorical predictors. Bayesian Anal 2019; 14: 341–369.

[42] Malsiner-Walli G Pauger D Wagner H. Effect fusion using model-based clustering. Stat Model 2018; 18: 175–196.

[43] Wagner H Pauger D. Discussion: Bayesian regularization and effect smoothing for categorical predictors. Stat Model 2016; 16: 220–227.

[44] Eilers PH Marx BD. Flexible smoothing using B-splines and penalized likelihood. Stat Sci 1996; 11: 89–121.

[45] Eilers PH Marx BD. Practical Smoothing: The Joys of P-splines. Cambridge, UK: Cambridge University Press, 2021.

[46] Hofner B Hothorn T Kneib T, et al. A framework for unbiased model selection based on boosting. J Comput Graph Stat 2011; 20: 956–971.

[47] Köllmann C Bornkamp B Ickstadt K. Unimodal regression using Bernstein–Schoenberg splines and penalties. Biometrics 2014; 70: 783–793. 24975523 10.1111/biom.12193

[48] Fahrmeir L Kneib T Lang S. Penalized structured additive regression for space-time data: a Bayesian perspective. Stat Sin 2004; 14: 731–761.

[49] Fahrmeir L Kneib T. Bayesian smoothing and regression for longitudinal, spatial and event history data. New York: Oxford University Press, 2011.

[50] Spiegel E Kneib T Otto-Sobotka F. Generalized additive models with flexible response functions. Stat Comput 2019; 29: 123–138.

[51] Fahrmeir L Kneib T Lang S, et al. Regression. 2nd ed. Berlin: Springer, 2021.

[52] R Core Team. *R: A Language and Environment for Statistical Computing*. R Foundation for Statistical Computing, Vienna, Austria, 2018. https://www.R-project.org/.

[53] Friedman J Hastie T Tibshirani R. Regularization paths for generalized linear models via coordinate descent. J Stat Softw 2010; 33: 1–22. 20808728 PMC2929880

[54] Goeman JJ Meijer RJ Chaturvedi N. *Penalized: L1 (lasso and fused lasso) and L2 (ridge) penalized estimation in GLMs and in the Cox model*, 2018. R package version 0.9-51.

[55] Freund Y Schapire R. Experiments with a new boosting algorithm. In *Proceedings of the Thirteenth International Conference on Machine Learning Theory*. San Francisco, CA: San Francisco: Morgan Kaufmann Publishers Inc., pp. 148–156.

[56] Friedman JH Hastie T Tibshirani R. Additive logistic regression: a statistical view of boosting (with discussion). Ann Stat 2000; 28: 337–407.

[57] Friedman JH. Greedy function approximation: a gradient boosting machine. Ann Stat 2001; 29: 1189–1232.

[58] Mayr A Binder H Gefeller O, et al. Extending statistical boosting - an overview of recent methodological developments. Methods Inf Med 2014; 53: 428–435. 25112429 10.3414/ME13-01-0123

[59] Hothorn T Buehlmann P Kneib T, et al. *mboost: Model-Based Boosting*, 2021. https://CRAN.R-project.org/package=mboost. R package version 2.9-5.

[60] Greenwell B Boehmke B Cunningham J, et al. *gbm: Generalized Boosted Regression Models*, 2020. https://CRAN.R-project.org/package=gbm. R package version 2.1.8.

[61] Chen T He T Benesty M, et al. Xgboost: extreme gradient boosting. R package version 04-2 2015; 1: 1–4.

[62] Breiman L Friedman JH Olshen RA, et al. Classification and regression trees. Monterey, CA: Wadsworth, 1984.

[63] Goodfellow I Bengio Y Courville A. Deep learning. Cambridge, MA: MIT press, 2016.

[64] Fritsch S Guenther F Wright MN. *neuralnet*. Training of Neural Networks, 2019. https://CRAN.R-project.org/package=neuralnet. R package version 1.44.2.

[65] Rong X. *deepnet: deep learning toolkit in R*, 2014. https://CRAN.R-project.org/package=deepnet. R package version 0.2.

[66] Allaire J Chollet F. *keras: R Interface to ’Keras’*, 2021. https://CRAN.R-project.org/package=keras. R package version 2.6.1.

[67] Probst P Boulesteix AL. To tune or not to tune the number of trees in random forest?. J Mach Learn Res 2017; 18: 1–18.

[68] Wright MN Ziegler A. ranger: a fast implementation of random forests for high dimensional data in C++ and R. J Stat Softw 2017; 77: 1–17.

[69] Hothorn T Hornik K Zeileis A. Unbiased recursive partitioning: a conditional inference framework. J Comput Graph Stat 2006; 15: 651–674.

[70] Hothorn T Bühlmann P Dudoit S, et al. Survival ensembles. Biostatistics 2006; 7: 355–373. 16344280 10.1093/biostatistics/kxj011

[71] Strobl C Boulesteix AL Zeileis A, et al. Bias in random forest variable importance measures: illustrations, sources and a solution. BMC Bioinformatics 2007; 8: 25. 17254353 10.1186/1471-2105-8-25 PMC1796903

[72] Strobl C Boulesteix AL Kneib T, et al. Conditional variable importance for random forests. BMC Bioinformatics 2008; 9: 307. 18620558 10.1186/1471-2105-9-307 PMC2491635

[73] Lang M Binder M Richter J, et al. mlr3: a modern object-oriented machine learning framework in R. J Open Source Softw 2019; 4: 1903. DOI: 10.21105/joss.01903. https://joss.theoj.org/papers/10.21105/joss.01903.

[74] Raftery A Hoeting J Volinsky C, et al. *BMA: Bayesian Model Averaging*, 2021. https://CRAN.R-project.org/package=BMA. R package version 3.18.15.

[75] Zeugner S Feldkircher M. Bayesian model averaging employing fixed and flexible priors: the BMS package for R. J Stat Softw 2015; 68: 1–37.

[76] Bartoǹ K. *MuMIn: Multi-Model Inference*, 2020. https://CRAN.R-project.org/package=MuMIn. R package version 1.43.17.

[77] Thomas J Hepp T Mayr A, et al. Probing for sparse and fast variable selection with model-based boosting. Comput Math Methods Med 2017; 1421409: 1–8. 10.1155/2017/1421409 PMC5555005 28831289

[78] Srivastava N Hinton G Krizhevsky A, et al. Dropout: a simple way to prevent neural networks from overfitting. J Mach Learn Res 2014; 15: 1929–1958.

[79] Saifi S. Prostate cancer dataset. https://www.kaggle.com/sajidsaifi/prostate-cancer, 2021. Accessed November 11th, 2021.

[80] Groll A Hastie T Tutz G. Selection of effects in cox frailty models by regularization methods. Biometrics 2017; 73: 846–856. 28085181 10.1111/biom.12637 PMC6261611

[81] Tutz G Binder H. Generalized additive modeling with implicit variable selection by likelihood-based boosting. Biometrics 2006; 62: 961–971. 17156269 10.1111/j.1541-0420.2006.00578.x

[82] Hofner B Hothorn T Kneib T. Variable selection and model choice in structured survival models. Comput Stat 2013; 28: 1079–1101.

[83] Schmid M Hothorn T. Boosting additive models using component-wise p-splines. Comput Stat Data Anal 2008; 53: 298–311.

[84] Klein N Carlan M Kneib T, et al. Bayesian effect selection in structured additive distributional regression models. Bayesian Anal 2021; 16: 545–573.

[85] Scheipl F Fahrmeir L Kneib T. Spike-and-slab priors for function selection in structured additive regression models. J Am Stat Assoc 2012; 107: 1518–1532.

[86] Diggle PJ Liang KY Zeger SL. Analysis of longitudinal data. Oxford: Oxford University Press, 1994.

[87] Hastie T Tibshirani R Friedman J. *The elements of statistical learning*. Springer series in statistics New York, 2001.

[88] Stamey TA Kabalin JN McNeal JE, et al. Prostate specific antigen in the diagnosis and treatment of adenocarcinoma of the prostate ii radical prostatectomy treated patients. J Urol 1989; 141: 1076–1083. 2468795 10.1016/s0022-5347(17)41175-x

[89] Varges D Manthey H Heinemann U, et al. Doxycycline in early cjd: a double-blinded randomised phase ii and observational study. J Neurol Neurosurg Psychiatry 2017; 88: 119–125. 27807198 10.1136/jnnp-2016-313541 PMC5284486

[90] Gross O Tönshoff B Weber LT, et al. A multicenter, randomized, placebo-controlled, double-blind phase 3 trial with open-arm comparison indicates safety and efficacy of nephroprotective therapy with ramipril in children with Alport’s syndrome. Kidney Int 2020; 97: 1275–1286. 32299679 10.1016/j.kint.2019.12.015

[91] Kruegel J Rubel D Gross O. Alport syndrome–insights from basic and clinical research. Nat Rev Nephrol 2013; 9: 170. 23165304 10.1038/nrneph.2012.259

[92] Rücker G Reiser V Motschall E, et al. Boosting qualifies capture–recapture methods for estimating the comprehensiveness of literature searches for systematic reviews. J Clin Epidemiol 2011; 64: 1364–1372. 21684116 10.1016/j.jclinepi.2011.03.008

[93] Website of SAS Institute. https://sas.com. Accessed on June 29th, 2022.

[94] Riley RD Snell KI Martin GP, et al. Penalization and shrinkage methods produced unreliable clinical prediction models especially when sample size was small. J Clin Epidemiol 2021; 132: 88–96. 33307188 10.1016/j.jclinepi.2020.12.005 PMC8026952

[95] Sauerbrei W Abrahamowicz M Altman DG, et al. Strengthening analytical thinking for observational studies: the stratos initiative. Stat Med 2014; 33: 5413–5432. 25074480 10.1002/sim.6265 PMC4320765

[96] Sauerbrei W Perperoglou A Schmid M, et al. State of the art in selection of variables and functional forms in multivariable analysis-outstanding issues. Diagn Progn Res 2020; 4: 3. 32266321 10.1186/s41512-020-00074-3 PMC7114804

