# Supplementary material for: Regularization approaches in clinical biostatistics: A review of methods and their applications
Source: Stat Methods Med Res. 2022 Nov 16;32(2):425–40. doi: 10.1177/09622802221133557 (PMC9896544; doi:10.1177/09622802221133557)
Supplement: sj-pdf-1-smm-10.1177_09622802221133557 - Supplemental material for Regularization approaches in clinical biostatistics: A review of methods and their applications [file sj-pdf-1-smm-10.1177_09622802221133557.pdf]

Supplemental Material to: Regularization approaches in clinical biostatistics - A review of methods and their applications

Sarah Friedrich, Andreas Groll, Katja Ickstadt, Thomas Kneib,
Markus Pauly, Jörg Rahnenführer, Tim Friede

This supplemental material to the paper "Regularization approaches in clinical biostatistics: A review of methods and their application" contains an example implementation of different regularization approaches applied to a data set on prostate cancer. The data set can be obtained from kaggle (https://www.kaggle.com /sajidsaifi/prostate-cancer).

Moreover, it contains additional results of the literature review as well as an overview of the number of CRAN downloads for each of the R-packages mentioned in the paper.

The authors would like to thank Daniel Klippert for the preparation of the data examples and the corresponding R code.

1 Additional results of the literature review

Table 1: Study characteristics of the studies included in the literature review according to journal. For detailed information on the regularization used, see Table 2 in the main manuscript. Numbers are $n(\%)$ unless otherwise stated. IQR = Interquartile range, NA = no information provided

		JAMA	NEJM	BMJ
Sample size	median (IQR)	1796 [300; 27730]	425 [144; 1660]	23416 [745; 500590]
Software used				
	R	27 (27%)	22 (13%)	37 (32%)
	SAS	50 (50%)	60 (37%)	26 (22%)
	Stata	28 (28 %)	11 (7%)	36 (31%)
	SPSS	6 (6%)	9 (6%)	9 (8%)
	Other	0 (0 %)	7 (4%)	7 (6%)
	NA	7 (7%)	68 (42%)	16 (14%)
Regularization used				
	None	62 (62%)	121 (74%)	70 (60%)
	Random effects	35 (35%)	31 (19%)	38 (33%)
	Other	6 (6%)	14 (9%)	14 (12%)

```
knitr::include_graphics("PRISMA.pdf")
```

2 Analysis of the prostate cancer data set

In this section, we analyse the prostate cancer data set from kaggle by applying some of the regularization approaches discussed in the paper.

2.1 Data Preparation

PRISMA 2020 flow diagram for new systematic reviews which included searches of databases and registers only

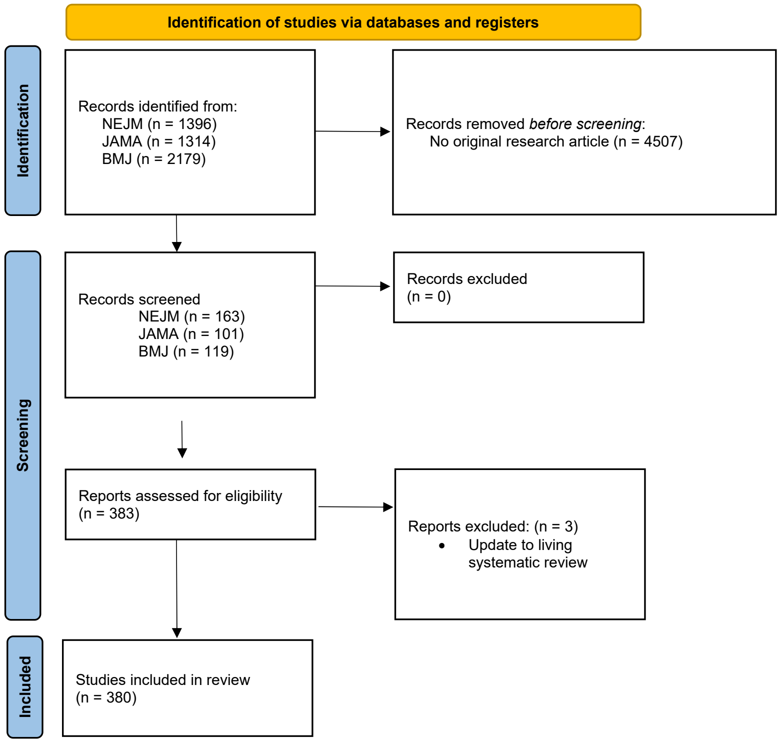

From: Page MJ, McKenzie JE, Bossuyt PM, Boutron I, Hoffmann TC, Mulrow CD, et al. The PRISMA 2020 statement: an updated guideline for reporting systematic reviews. BMJ 2021;372:n71. doi: 10.1136/bmj.n71

For more information, visit: http://www.prisma-statement.org/

Figure 1: PRISMA Flow Chart of the literature review

```
options(warn=-1)
dim(df)
```

```
## [1] 100  10
```

```
df$id <- NULL
df$diagnosis_result <- ifelse(df$diagnosis_result == "M", 1, 0)
df$diagnosis_result <- as.factor(df$diagnosis_result)
```

```
iter = 100
```

2.2 Logistic Regression

For comparison, we first fit a simple logistic regression model to the data. Here, we wish to predict the diagnosis result based on the tumor characteristics included in the data set. To make the code comparable, we make use of the mlr3-package for all methods considered here.

```
library("mlr3")
library("mlr3verse")
library("mlr3tuning")

requireNamespace("lgr")
logger = lgr::get_logger("mlr3")
logger$set_threshold("warn")
lgr::get_logger("bbotk")$set_threshold("warn")
```

```
task = TaskClassif$new(id = "LogReg", backend = df, target = "diagnosis_result")
learner = lrn("classif.log_reg", predict_type = "prob")

set.seed(421)

rr_list <- list()
resample <- rsmp("holdout", ratio = 7/10)
for(i in 1:iter){
  rr_list[[i]] <- resample(task, learner, resample, store_models = T)
}

df_log_reg <- data.frame("MCE" = sapply(1:iter, function(x)
  rr_list[[x]]$aggregate(msr("classif.ce"))), "AUC" = sapply(1:iter, function(x)
    rr_list[[x]]$aggregate(msr("classif.auc"))))
mean(df_log_reg$MCE)
```

```
## [1] 0.1833333
```

```
mean(df_log_reg$AUC)
```

```
## [1] 0.8836972
```

2.3 Logistic Regression with two-way interactions

To provide a comprehensive picture of the situation, we also consider the logistic regression model with two-way interactions.

```
library("tidymodels")
library("pROC")
```

```r
set.seed(421)
logreg_full_twoway_mce = numeric(iter)
logreg_full_twoway_auc = numeric(iter)
```

```r
for(i in 1:iter){
  split_breast= initial_split(data = df, prop = 0.7)
  train_breast = training(split_breast)
  test_breast = testing(split_breast)
  model=glm(diagnosis_result ~ .^2, family = binomial(link = "logit"), train_breast)
  preds = predict(model, test_breast, type = "response")
  predicted.classes = ifelse(preds > 0.5, 1, 0)
  logreg_full_twoway_mce[i]=mean(predicted.classes != test_breast$diagnosis_result)
  logreg_full_twoway_auc[i]=roc(test_breast$diagnosis_result,preds, quiet=T)$auc
}
df_log_reg_full_twoway <- data.frame("MCE" = logreg_full_twoway_mce,
                                     "AUC" = logreg_full_twoway_auc)
df_log_reg_full_twoway
```

```
##           MCE       AUC
## 1   0.3333333 0.6818182
## 2   0.5333333 0.4950000
## 3   0.2333333 0.7828054
## 4   0.3000000 0.7589286
## 5   0.4000000 0.6365741
## 6   0.3666667 0.4813665
## 7   0.2333333 0.7700000
## 8   0.4000000 0.5568182
## 9   0.3333333 0.6800000
## 10  0.2666667 0.7407407
## 11  0.3666667 0.6527778
## 12  0.4000000 0.6291866
## 13  0.3333333 0.6428571
## 14  0.3000000 0.6477273
## 15  0.5666667 0.5502392
## 16  0.4000000 0.6375661
## 17  0.2333333 0.7513228
## 18  0.2333333 0.6925466
## 19  0.4333333 0.5625000
## 20  0.3666667 0.6574074
## 21  0.3333333 0.6435185
## 22  0.3666667 0.6643519
## 23  0.4333333 0.6607143
## 24  0.3333333 0.6711111
## 25  0.3333333 0.6111111
## 26  0.4000000 0.6878307
## 27  0.2666667 0.7460317
## 28  0.5333333 0.5520362
## 29  0.3666667 0.6517857
## 30  0.1666667 0.8194444
## 31  0.2333333 0.6349206
## 32  0.3666667 0.6534091
## 33  0.3333333 0.6647727
## 34  0.4000000 0.6651584
## 35  0.5666667 0.5113122
```

```
## 36  0.1666667 0.8303167
## 37  0.2333333 0.7763975
## 38  0.3000000 0.6470588
## 39  0.3333333 0.6225000
## 40  0.4000000 0.6984127
## 41  0.5666667 0.4531250
## 42  0.2666667 0.6903409
## 43  0.3333333 0.6968326
## 44  0.3333333 0.7075893
## 45  0.3666667 0.6086957
## 46  0.3333333 0.6897321
## 47  0.4333333 0.5625000
## 48  0.4000000 0.6289593
## 49  0.5333333 0.4950000
## 50  0.3000000 0.7901786
## 51  0.1666667 0.8102679
## 52  0.3000000 0.7352941
## 53  0.3666667 0.6180556
## 54  0.4333333 0.5358852
## 55  0.2333333 0.8333333
## 56  0.5000000 0.6079545
## 57  0.4666667 0.5820106
## 58  0.2666667 0.7450000
## 59  0.4666667 0.5340909
## 60  0.3333333 0.7453704
## 61  0.4666667 0.5600000
## 62  0.3333333 0.6961722
## 63  0.3000000 0.6898148
## 64  0.3333333 0.5750000
## 65  0.4000000 0.5952381
## 66  0.3333333 0.7050000
## 67  0.3666667 0.6435185
## 68  0.4000000 0.5750000
## 69  0.3666667 0.6650718
## 70  0.4000000 0.6116071
## 71  0.5000000 0.5429864
## 72  0.3333333 0.7777778
## 73  0.2666667 0.7950000
## 74  0.2000000 0.8122172
## 75  0.2333333 0.6732955
## 76  0.2333333 0.8095238
## 77  0.3666667 0.6473214
## 78  0.2333333 0.8133971
## 79  0.3333333 0.7250000
## 80  0.4333333 0.5079365
## 81  0.4333333 0.5837321
## 82  0.3333333 0.7053571
## 83  0.4000000 0.5050000
## 84  0.1666667 0.8438914
## 85  0.3333333 0.6851852
## 86  0.2666667 0.7285068
## 87  0.4000000 0.6225000
## 88  0.3000000 0.7037037
## 89  0.3666667 0.5701357
```

```
## 90  0.3000000 0.6698565
## 91  0.3666667 0.6844444
## 92  0.5000000 0.5574163
## 93  0.3333333 0.6650000
## 94  0.4666667 0.5550239
## 95  0.3666667 0.7009569
## 96  0.3333333 0.6746411
## 97  0.3000000 0.7100000
## 98  0.2666667 0.7354497
## 99  0.2333333 0.7655502
## 100 0.2000000 0.8597285
```

```
mean(df_log_reg_full_twoway$MCE)
```

```
## [1] 0.348
```

```
mean(df_log_reg_full_twoway$AUC)
```

```
## [1] 0.6643755
```

2.4 Logistic Regression with all two-way interactions involving 'compactness'

Similarly, we can consider only two-way interactions which involve the univariately significant variable 'compactness':

```
# determine significant variables:
full_model = glm(diagnosis_result ~ ., family=binomial(link="logit"),data=df)
summary(full_model)
```

```
##
## Call:
## glm(formula = diagnosis_result ~ ., family = binomial(link = "logit"),
##     data = df)
##
## Deviance Residuals:
##     Min       1Q   Median       3Q      Max
## -3.4909  -0.3416   0.1959   0.4555   1.5749
##
## Coefficients:
##                     Estimate Std. Error z value Pr(>|z|)
## (Intercept)        8.783e-01  1.470e+01   0.060   0.9524
## radius            -2.006e-02  6.969e-02  -0.288   0.7735
## texture            7.915e-02  6.970e-02   1.136   0.2561
## perimeter          9.481e-02  2.057e-01   0.461   0.6448
## area              -3.468e-03  1.324e-02  -0.262   0.7934
## smoothness        -2.014e+01  2.915e+01  -0.691   0.4897
## compactness        4.622e+01  2.324e+01   1.989   0.0467 *
## symmetry          -4.738e+00  1.911e+01  -0.248   0.8042
## fractal_dimension -1.615e+02  1.271e+02  -1.270   0.2040
## ---
## Signif. codes:  0 '***' 0.001 '**' 0.01 '*' 0.05 '.' 0.1 ' ' 1
##
## (Dispersion parameter for binomial family taken to be 1)
##
##     Null deviance: 132.81  on 99  degrees of freedom
## Residual deviance:  66.24  on 91  degrees of freedom
```

```
## AIC: 84.24
##
## Number of Fisher Scoring iterations: 6
```

```
logreg_compactness_mce = numeric(iter)
logreg_compactness_auc = numeric(iter)
```

```
set.seed(421)

for(i in 1:iter){
  split_breast= initial_split(data = df, prop = 0.7)
  train_breast = training(split_breast)
  test_breast = testing(split_breast)
  model=glm(diagnosis_result ~ .*compactness, family = binomial(link = "logit"), train_breast)
  preds = predict(model, test_breast, type = "response")
  predicted.classes = ifelse(preds > 0.5, 1, 0)
  logreg_compactness_mce[i]=mean(predicted.classes != test_breast$diagnosis_result)
  logreg_compactness_auc[i]=roc(test_breast$diagnosis_result,preds)$auc
}
df_log_reg_compactness <- data.frame("MCE" = logreg_compactness_mce,
                                     "AUC" = logreg_compactness_auc)
df_log_reg_compactness
```

```
##             MCE       AUC
## 1   0.23333333 0.8899522
## 2   0.30000000 0.6500000
## 3   0.20000000 0.7986425
## 4   0.20000000 0.8526786
## 5   0.23333333 0.8148148
## 6   0.33333333 0.7391304
## 7   0.20000000 0.8900000
## 8   0.30000000 0.7215909
## 9   0.33333333 0.6550000
## 10  0.36666667 0.7037037
## 11  0.10000000 0.8981481
## 12  0.23333333 0.7990431
## 13  0.26666667 0.8169643
## 14  0.16666667 0.7500000
## 15  0.30000000 0.7559809
## 16  0.06666667 0.9894180
## 17  0.23333333 0.7380952
## 18  0.23333333 0.8633540
## 19  0.16666667 0.8500000
## 20  0.13333333 0.8796296
## 21  0.26666667 0.7638889
## 22  0.20000000 0.8750000
## 23  0.30000000 0.7142857
## 24  0.26666667 0.8622222
## 25  0.20000000 0.8201058
## 26  0.23333333 0.7354497
## 27  0.06666667 0.9894180
## 28  0.13333333 0.8733032
## 29  0.26666667 0.7834821
## 30  0.23333333 0.8750000
## 31  0.23333333 0.6957672
```

```
## 32  0.10000000 0.9375000
## 33  0.26666667 0.6619318
## 34  0.20000000 0.7963801
## 35  0.20000000 0.8212670
## 36  0.30000000 0.7194570
## 37  0.20000000 0.7515528
## 38  0.16666667 0.8597285
## 39  0.16666667 0.8400000
## 40  0.13333333 0.8994709
## 41  0.40000000 0.6919643
## 42  0.20000000 0.8210227
## 43  0.33333333 0.7828054
## 44  0.43333333 0.5870536
## 45  0.26666667 0.8012422
## 46  0.20000000 0.9375000
## 47  0.36666667 0.6830357
## 48  0.16666667 0.8416290
## 49  0.40000000 0.5875000
## 50  0.26666667 0.7500000
## 51  0.30000000 0.7232143
## 52  0.16666667 0.8868778
## 53  0.13333333 0.9629630
## 54  0.33333333 0.6363636
## 55  0.10000000 0.9312169
## 56  0.23333333 0.8181818
## 57  0.20000000 0.7777778
## 58  0.30000000 0.6750000
## 59  0.23333333 0.7670455
## 60  0.20000000 0.8287037
## 61  0.23333333 0.7950000
## 62  0.20000000 0.8516746
## 63  0.20000000 0.8125000
## 64  0.43333333 0.6000000
## 65  0.33333333 0.6984127
## 66  0.46666667 0.5125000
## 67  0.26666667 0.7546296
## 68  0.23333333 0.7300000
## 69  0.30000000 0.7320574
## 70  0.26666667 0.7656250
## 71  0.26666667 0.7511312
## 72  0.36666667 0.7513228
## 73  0.23333333 0.7750000
## 74  0.16666667 0.8619910
## 75  0.26666667 0.8181818
## 76  0.16666667 0.9100529
## 77  0.33333333 0.7589286
## 78  0.10000000 0.9808612
## 79  0.16666667 0.8900000
## 80  0.10000000 0.8730159
## 81  0.33333333 0.7224880
## 82  0.36666667 0.7232143
## 83  0.10000000 0.9400000
## 84  0.13333333 0.9140271
## 85  0.26666667 0.8148148
```

```
## 86  0.16666667 0.8642534
## 87  0.16666667 0.9000000
## 88  0.33333333 0.7314815
## 89  0.33333333 0.6289593
## 90  0.23333333 0.7416268
## 91  0.30000000 0.7688889
## 92  0.26666667 0.6004785
## 93  0.20000000 0.8800000
## 94  0.30000000 0.7511962
## 95  0.33333333 0.6626794
## 96  0.16666667 0.8612440
## 97  0.26666667 0.7750000
## 98  0.16666667 0.7989418
## 99  0.20000000 0.8133971
## 100 0.16666667 0.8597285
```

2.5 Ridge Regression

We start with the penalization approaches described in Section 2.1 of the paper. In a first step, ridge regression, i.e., an L2 penalty is considered.

```
df$radius <- (df$radius - mean(df$radius))/sd(df$radius)
df$texture <- (df$texture - mean(df$texture))/sd(df$texture)
df$perimeter <- (df$perimeter - mean(df$perimeter))/sd(df$perimeter)
df$area <- (df$area - mean(df$area))/sd(df$area)
df$smoothness <- (df$smoothness - mean(df$smoothness))/sd(df$smoothness)
df$compactness <- (df$compactness - mean(df$compactness))/sd(df$compactness)
df$symmetry <- (df$symmetry - mean(df$symmetry))/sd(df$symmetry)
df$fractal_dimension <- (df$fractal_dimension -
                           mean(df$fractal_dimension))/sd(df$fractal_dimension)

measure <- msr("classif.logloss")
terminator= trm("none")
param_set = ps(
  lambda = p_dbl(log(1), log(200), trafo = function(x) exp(x)-0.999))

ridge_ce <- rep(0, iter)
ridge_auc <- rep(0, iter)
lambda_ridge  <- rep(0, iter)
for(i in 1:iter){
  train_set = sample(1:length(df[,1]), 0.7 * length(df[,1]))
  test_set = setdiff(1:length(df[,1]), train_set)
  task_tune = TaskClassif$new(id = paste("tune", i), backend = df[train_set,],
                              target = "diagnosis_result")
  resample_tune = rsmp("cv", folds = 10)
  learner_tune = lrn("classif.glmnet", predict_type = "prob", alpha = 0)
  tuner <- tnr("grid_search", resolution = 30)

  instance_tune <- TuningInstanceSingleCrit$new(task = task_tune,
                     learner = learner_tune, resampling = resample_tune,
                    measure =  measure, terminator =  terminator,
                    search_space = param_set, store_models = TRUE)
  tuner$optimize(instance_tune)
  learner_tune = lrn("classif.glmnet", predict_type = "prob", alpha = 0)
```

```
  learner_tune$param_set$values$lambda <- instance_tune$result_x_domain$lambda
  lambda_ridge[i] <- instance_tune$result_x_domain$lambda
  learner_tune$train(task_tune)
  prediction = learner_tune$predict_newdata(df[test_set,])
  ridge_ce[i] <- prediction$score(msr("classif.ce"))
  ridge_auc[i] <- prediction$score(msr("classif.auc"))
}
df_log_reg_ridge <- data.frame("MCE" = ridge_ce, "AUC" = ridge_auc,
                               "Lambda" = lambda_ridge)
df_log_reg_ridge
```

```
##             MCE       AUC    Lambda
## 1   0.06666667 0.8564815 0.2014549
## 2   0.13333333 0.9638009 0.2014549
## 3   0.10000000 0.9521531 0.2014549
## 4   0.06666667 0.9760766 0.2014549
## 5   0.16666667 0.9185520 0.2014549
## 6   0.13333333 0.8516746 0.2014549
## 7   0.20000000 0.8560000 0.2014549
## 8   0.16666667 0.9350000 0.2014549
## 9   0.06666667 0.9875776 0.2014549
## 10  0.10000000 0.9305556 0.2014549
## 11  0.13333333 0.8657407 0.2014549
## 12  0.13333333 0.9431818 0.2014549
## 13  0.23333333 0.8050000 0.2014549
## 14  0.26666667 0.7873303 0.2014549
## 15  0.10000000 0.8516746 0.2014549
## 16  0.10000000 0.9732143 0.2014549
## 17  0.06666667 0.9819005 0.2014549
## 18  0.20000000 0.8194444 0.0010000
## 19  0.13333333 0.9400000 0.2014549
## 20  0.20000000 0.9130435 0.2014549
## 21  0.06666667 0.8468900 0.2014549
## 22  0.23333333 0.8300000 0.2014549
## 23  0.20000000 0.8181818 0.2014549
## 24  0.06666667 0.9850000 0.2014549
## 25  0.13333333 0.8300000 0.2014549
## 26  0.10000000 0.9907407 0.2014549
## 27  0.13333333 0.9675926 0.2014549
## 28  0.16666667 0.9351852 0.2014549
## 29  0.10000000 0.9150000 0.2014549
## 30  0.20000000 0.8842593 0.2014549
## 31  0.20000000 0.8616071 0.2014549
## 32  0.10000000 0.9377990 0.2014549
## 33  0.13333333 0.9090909 0.2014549
## 34  0.16666667 0.8660287 0.0010000
## 35  0.13333333 0.9107143 0.2014549
## 36  0.16666667 0.8133971 0.0010000
## 37  0.16666667 0.9366516 0.2014549
## 38  0.03333333 0.9735450 0.2014549
## 39  0.16666667 0.8611111 0.2014549
## 40  0.13333333 0.8981481 0.2014549
## 41  0.16666667 0.9111111 0.2014549
## 42  0.13333333 0.7894737 0.2014549
```

```
## 43  0.16666667 0.9955357 0.2014549
## 44  0.13333333 0.8981481 0.2014549
## 45  0.10000000 0.8633540 0.2014549
## 46  0.10000000 0.8803828 0.2014549
## 47  0.16666667 0.9592760 0.2014549
## 48  0.10000000 0.8947368 0.2014549
## 49  0.06666667 0.9330357 0.2014549
## 50  0.16666667 0.9378882 0.2014549
## 51  0.16666667 0.8851675 0.2014549
## 52  0.20000000 0.9017857 0.2014549
## 53  0.26666667 0.8295455 0.2014549
## 54  0.06666667 1.0000000 0.2014549
## 55  0.16666667 0.8100000 0.2014549
## 56  0.13333333 0.9375000 0.2014549
## 57  0.13333333 0.9665072 0.2014549
## 58  0.16666667 0.8624339 0.2014549
## 59  0.10000000 0.8700000 0.2014549
## 60  0.10000000 0.8835979 0.2014549
## 61  0.16666667 0.8400000 0.0010000
## 62  0.20000000 0.9100000 0.2014549
## 63  0.23333333 0.8437500 0.2014549
## 64  0.16666667 0.8564593 0.2014549
## 65  0.06666667 0.9861111 0.2014549
## 66  0.16666667 0.8693182 0.2014549
## 67  0.10000000 0.9351852 0.2014549
## 68  0.20000000 0.9004525 0.2014549
## 69  0.16666667 0.8086124 0.2014549
## 70  0.03333333 1.0000000 0.2014549
## 71  0.06666667 0.9166667 0.2014549
## 72  0.10000000 0.9773756 0.2014549
## 73  0.20000000 0.9107143 0.2014549
## 74  0.16666667 0.8240741 0.2014549
## 75  0.13333333 0.9138756 0.2014549
## 76  0.06666667 0.9773756 0.2014549
## 77  0.13333333 0.9473684 0.2014549
## 78  0.10000000 0.9244444 0.2014549
## 79  0.16666667 0.8941799 0.2014549
## 80  0.23333333 0.8616071 0.2014549
## 81  0.10000000 0.9120370 0.2014549
## 82  0.13333333 0.8600000 0.2014549
## 83  0.13333333 0.9629630 0.2014549
## 84  0.16666667 0.9100000 0.2014549
## 85  0.16666667 0.8409091 0.0010000
## 86  0.10000000 0.8516746 0.2014549
## 87  0.10000000 0.9523810 0.2014549
## 88  0.06666667 0.9864253 0.2014549
## 89  0.13333333 0.9259259 0.2014549
## 90  0.33333333 0.8750000 0.2014549
## 91  0.10000000 0.9659091 0.2014549
## 92  0.10000000 0.9750000 0.2014549
## 93  0.10000000 0.8888889 0.2014549
## 94  0.03333333 0.9819005 0.2014549
## 95  0.03333333 0.9768519 0.2014549
## 96  0.13333333 0.8352273 0.2014549
```

```
## 97  0.13333333 0.8850000 0.2014549
## 98  0.13333333 0.9425837 0.2014549
## 99  0.20000000 0.8101852 0.2014549
## 100 0.16666667 0.9241071 0.2014549
```

```
mean(df_log_reg_ridge$MCE)
```

```
## [1] 0.138
```

```
mean(df_log_reg_ridge$AUC)
```

```
## [1] 0.9044936
```

2.6 LASSO

We now consider an L1 penalty, which additionally to shrinkage also performs variable selection.

```
lasso_ce <- rep(0, iter)
lasso_auc <- rep(0, iter)
lambda_lasso <- rep(0, iter)
for(i in 1:iter){
  train_set = sample(1:length(df[,1]), 0.7 * length(df[,1]))
  test_set = setdiff(1:length(df[,1]), train_set)
  task_tune = TaskClassif$new(id = paste("tune", i), backend = df[train_set,],
                              target = "diagnosis_result")
  resample_tune = rsmp("cv", folds = 10)
  learner_tune = lrn("classif.glmnet", predict_type = "prob", alpha = 1)
  tuner <- tnr("grid_search", resolution = 30)

  instance_tune <- TuningInstanceSingleCrit$new(task = task_tune,
                              learner = learner_tune, resampling = resample_tune,
                              measure =  measure, terminator =  terminator,
                              search_space = param_set, store_models = TRUE)
  tuner$optimize(instance_tune)

  learner_tune = lrn("classif.glmnet", predict_type = "prob", alpha = 1)

  learner_tune$param_set$values$lambda <- instance_tune$result_x_domain$lambda
  lambda_lasso[i] <- instance_tune$result_x_domain$lambda
  learner_tune$train(task_tune)

  prediction = learner_tune$predict_newdata(df[test_set,])
  lasso_ce[i] <- prediction$score(msr("classif.ce"))
  lasso_auc[i] <- prediction$score(msr("classif.auc"))
}
df_log_reg_lasso <- data.frame("MCE" = lasso_ce, "AUC" = lasso_auc,
                               "Lambda" = lambda_lasso)
df_log_reg_lasso
```

```
##            MCE       AUC    Lambda
## 1   0.43333333 0.9864253 0.2014549
## 2   0.26666667 0.8642534 0.2014549
## 3   0.26666667 0.9377990 0.2014549
## 4   0.13333333 0.9431818 0.2014549
## 5   0.10000000 0.9841270 0.2014549
## 6   0.56666667 0.9049774 0.2014549
```

```
## 7   0.13333333 0.9312169 0.2014549
## 8   0.46666667 0.8705357 0.2014549
## 9   0.20000000 0.7671958 0.0010000
## 10  0.13333333 0.9440994 0.2014549
## 11  0.23333333 0.8923445 0.2014549
## 12  0.16666667 0.9650000 0.0010000
## 13  0.16666667 0.7942584 0.0010000
## 14  0.26666667 0.8981481 0.2014549
## 15  0.40000000 0.8959276 0.2014549
## 16  0.36666667 0.8958333 0.2014549
## 17  0.23333333 0.8275000 0.2014549
## 18  0.10000000 0.9583333 0.0010000
## 19  0.36666667 0.9004525 0.2014549
## 20  0.43333333 0.7488889 0.2014549
## 21  0.16666667 0.8500000 0.0010000
## 22  0.26666667 0.8468900 0.2014549
## 23  0.36666667 0.9583333 0.2014549
## 24  0.20000000 0.9829545 0.2014549
## 25  0.16666667 0.9234450 0.0010000
## 26  0.23333333 0.8660287 0.2014549
## 27  0.03333333 1.0000000 0.0010000
## 28  0.23333333 0.7607656 0.0010000
## 29  0.13333333 0.9732143 0.0010000
## 30  0.43333333 0.9107143 0.2014549
## 31  0.20000000 0.8803828 0.2014549
## 32  0.13333333 0.9417989 0.2014549
## 33  0.26666667 0.9074074 0.2014549
## 34  0.26666667 0.8612440 0.2014549
## 35  0.13333333 0.9545455 0.2014549
## 36  0.26666667 0.9351852 0.2014549
## 37  0.06666667 1.0000000 0.0010000
## 38  0.10000000 0.8660287 0.0010000
## 39  0.43333333 0.8311111 0.2014549
## 40  0.16666667 0.8465608 0.0010000
## 41  0.00000000 1.0000000 0.2014549
## 42  0.33333333 0.8687783 0.2014549
## 43  0.26666667 0.9234450 0.2014549
## 44  0.26666667 0.8421053 0.2014549
## 45  0.36666667 0.8687783 0.2014549
## 46  0.36666667 0.9305556 0.2014549
## 47  0.23333333 0.8935185 0.2014549
## 48  0.26666667 0.7330317 0.0010000
## 49  0.30000000 0.7962963 0.0010000
## 50  0.26666667 0.7655502 0.0010000
## 51  0.10000000 0.9630682 0.2014549
## 52  0.23333333 0.8923445 0.2014549
## 53  0.36666667 0.9652778 0.2014549
## 54  0.13333333 0.9100000 0.0010000
## 55  0.13333333 0.8888889 0.2014549
## 56  0.16666667 0.9185520 0.0010000
## 57  0.13333333 0.8465909 0.2014549
## 58  0.43333333 0.9185520 0.2014549
## 59  0.20000000 0.8611111 0.0010000
## 60  0.33333333 0.9095023 0.2014549
```

```
## 61  0.30000000 0.9004630 0.2014549
## 62  0.16666667 0.8947368 0.2014549
## 63  0.33333333 0.8959276 0.2014549
## 64  0.33333333 0.9375000 0.2014549
## 65  0.30000000 0.9500000 0.2014549
## 66  0.46666667 0.8488889 0.2014549
## 67  0.36666667 0.7488688 0.2014549
## 68  0.26666667 0.8684211 0.2014549
## 69  0.30000000 0.8912037 0.2014549
## 70  0.53333333 0.9218750 0.2014549
## 71  0.20000000 0.9829545 0.2014549
## 72  0.26666667 0.9450000 0.2014549
## 73  0.26666667 0.7826087 0.2014549
## 74  0.40000000 0.9241071 0.2014549
## 75  0.33333333 0.8823529 0.2014549
## 76  0.23333333 0.8778281 0.2014549
## 77  0.20000000 0.8100000 0.0010000
## 78  0.13333333 0.9488636 0.2014549
## 79  0.36666667 0.8212670 0.2014549
## 80  0.33333333 0.8936652 0.2014549
## 81  0.46666667 0.9040179 0.2014549
## 82  0.30000000 0.9500000 0.2014549
## 83  0.13333333 0.8730159 0.2014549
## 84  0.23333333 0.9005682 0.2014549
## 85  0.20000000 0.9600000 0.2014549
## 86  0.23333333 0.9760766 0.2014549
## 87  0.30000000 0.8468900 0.2014549
## 88  0.13333333 0.8900000 0.2014549
## 89  0.16666667 0.8468900 0.0010000
## 90  0.43333333 0.8973214 0.2014549
## 91  0.30000000 0.9650000 0.2014549
## 92  0.43333333 0.8861607 0.2014549
## 93  0.23333333 0.8778281 0.0010000
## 94  0.16666667 0.8803828 0.0010000
## 95  0.36666667 0.8506787 0.2014549
## 96  0.10000000 0.9943182 0.2014549
## 97  0.40000000 0.8444444 0.2014549
## 98  0.16666667 0.8892045 0.2014549
## 99  0.43333333 0.9773756 0.2014549
## 100 0.20000000 0.9401914 0.2014549
```

```
mean(df_log_reg_lasso$MCE)
```

```
## [1] 0.261
```

```
mean(df_log_reg_lasso$AUC)
```

```
## [1] 0.8957995
```

```
## pdf
##   2
```

```
## pdf
##   2
```

2.7 Elastic Net

As a final penalization approach, an elastic net is implemented:

```
param_set = ps(
  lambda = p_dbl(log(1), log(200), trafo = function(x) exp(x)-0.999),
  alpha = p_dbl(0, 1)
)
elnet_ce <- rep(0, iter)
elnet_auc <- rep(0, iter)
lambda_elnet  <- rep(0, iter)
alpha_elnet <- rep(0,iter)
for(i in 1:iter){
  train_set = sample(1:length(df[,1]), 0.7 * length(df[,1]))
  test_set = setdiff(1:length(df[,1]), train_set)
  task_tune = TaskClassif$new(id = paste("tune", i), backend = df[train_set,],
                              target = "diagnosis_result")
  resample_tune = rsmp("cv", folds = 10)
  learner_tune = lrn("classif.glmnet", predict_type = "prob")

  tuner <- tnr("grid_search", resolution = 9)

  instance_tune <- TuningInstanceSingleCrit$new(task = task_tune,
                    learner = learner_tune, resampling = resample_tune,
                    measure =  measure, terminator =  terminator,
                    search_space = param_set, store_models = TRUE)
  tuner$optimize(instance_tune)

  learner_tune = lrn("classif.glmnet", predict_type = "prob")

  learner_tune$param_set$values$lambda <- instance_tune$result_x_domain$lambda
  learner_tune$param_set$values$alpha <- instance_tune$result$alpha
  lambda_elnet[i] <- instance_tune$result_x_domain$lambda
  alpha_elnet[i] <- instance_tune$result$alpha
  learner_tune$train(task_tune)

  prediction = learner_tune$predict_newdata(df[test_set,])
  elnet_ce[i] <- prediction$score(msr("classif.ce"))
  elnet_auc[i] <- prediction$score(msr("classif.auc"))
}
df_log_reg_elnet <- data.frame("MCE" = ridge_ce, "AUC" = ridge_auc,
                               "Lambda" = lambda_elnet, "Alpha" = alpha_elnet)
df_log_reg_elnet
```

```
##           MCE       AUC    Lambda Alpha
## 1  0.06666667 0.8564815 0.0010000 0.000
## 2  0.13333333 0.9638009 0.9402274 0.000
## 3  0.10000000 0.9521531 0.9402274 0.000
## 4  0.06666667 0.9760766 0.0010000 0.000
## 5  0.16666667 0.9185520 0.9402274 0.000
## 6  0.13333333 0.8516746 0.0010000 0.000
## 7  0.20000000 0.8560000 0.9402274 0.000
## 8  0.16666667 0.9350000 0.0010000 0.000
## 9  0.06666667 0.9875776 0.9402274 0.000
## 10 0.10000000 0.9305556 0.9402274 0.000
```

```
## 11  0.13333333 0.8657407 0.9402274 0.000
## 12  0.13333333 0.9431818 0.9402274 0.000
## 13  0.23333333 0.8050000 0.9402274 0.000
## 14  0.26666667 0.7873303 0.9402274 0.000
## 15  0.10000000 0.8516746 0.9402274 0.000
## 16  0.10000000 0.9732143 0.9402274 0.000
## 17  0.06666667 0.9819005 0.9402274 0.000
## 18  0.20000000 0.8194444 0.0010000 0.000
## 19  0.13333333 0.9400000 0.0010000 0.000
## 20  0.20000000 0.9130435 0.0010000 0.000
## 21  0.06666667 0.8468900 0.0010000 0.000
## 22  0.23333333 0.8300000 0.9402274 0.000
## 23  0.20000000 0.8181818 0.0010000 0.000
## 24  0.06666667 0.9850000 0.9402274 0.000
## 25  0.13333333 0.8300000 0.9402274 0.000
## 26  0.10000000 0.9907407 0.9402274 0.000
## 27  0.13333333 0.9675926 0.9402274 0.000
## 28  0.16666667 0.9351852 0.9402274 0.000
## 29  0.10000000 0.9150000 0.9402274 0.000
## 30  0.20000000 0.8842593 0.0010000 0.750
## 31  0.20000000 0.8616071 0.9402274 0.000
## 32  0.10000000 0.9377990 0.0010000 0.000
## 33  0.13333333 0.9090909 0.9402274 0.000
## 34  0.16666667 0.8660287 0.0010000 0.000
## 35  0.13333333 0.9107143 0.0010000 0.000
## 36  0.16666667 0.8133971 0.9402274 0.000
## 37  0.16666667 0.9366516 0.9402274 0.000
## 38  0.03333333 0.9735450 0.0010000 0.000
## 39  0.16666667 0.8611111 0.9402274 0.000
## 40  0.13333333 0.8981481 0.9402274 0.000
## 41  0.16666667 0.9111111 0.9402274 0.000
## 42  0.13333333 0.7894737 0.9402274 0.000
## 43  0.16666667 0.9955357 0.9402274 0.000
## 44  0.13333333 0.8981481 0.9402274 0.000
## 45  0.10000000 0.8633540 0.0010000 0.000
## 46  0.10000000 0.8803828 0.9402274 0.000
## 47  0.16666667 0.9592760 0.9402274 0.000
## 48  0.10000000 0.8947368 0.9402274 0.000
## 49  0.06666667 0.9330357 0.9402274 0.000
## 50  0.16666667 0.9378882 0.0010000 0.000
## 51  0.16666667 0.8851675 0.9402274 0.000
## 52  0.20000000 0.9017857 0.0010000 0.000
## 53  0.26666667 0.8295455 0.0010000 0.000
## 54  0.06666667 1.0000000 0.9402274 0.000
## 55  0.16666667 0.8100000 0.0010000 0.000
## 56  0.13333333 0.9375000 0.9402274 0.000
## 57  0.13333333 0.9665072 0.9402274 0.000
## 58  0.16666667 0.8624339 0.0010000 0.000
## 59  0.10000000 0.8700000 0.9402274 0.000
## 60  0.10000000 0.8835979 0.9402274 0.000
## 61  0.16666667 0.8400000 0.0010000 0.000
## 62  0.20000000 0.9100000 0.0010000 0.000
## 63  0.23333333 0.8437500 0.0010000 0.000
## 64  0.16666667 0.8564593 0.9402274 0.000
```

```
## 65  0.06666667 0.9861111 0.0010000 0.000
## 66  0.16666667 0.8693182 0.9402274 0.000
## 67  0.10000000 0.9351852 0.0010000 0.000
## 68  0.20000000 0.9004525 0.9402274 0.000
## 69  0.16666667 0.8086124 0.0010000 0.000
## 70  0.03333333 1.0000000 0.9402274 0.000
## 71  0.06666667 0.9166667 0.9402274 0.000
## 72  0.10000000 0.9773756 0.9402274 0.000
## 73  0.20000000 0.9107143 0.0010000 0.000
## 74  0.16666667 0.8240741 0.0010000 0.625
## 75  0.13333333 0.9138756 0.0010000 0.000
## 76  0.06666667 0.9773756 0.0010000 0.000
## 77  0.13333333 0.9473684 0.9402274 0.000
## 78  0.10000000 0.9244444 0.9402274 0.000
## 79  0.16666667 0.8941799 0.9402274 0.000
## 80  0.23333333 0.8616071 0.9402274 0.000
## 81  0.10000000 0.9120370 0.9402274 0.000
## 82  0.13333333 0.8600000 0.9402274 0.000
## 83  0.13333333 0.9629630 0.9402274 0.000
## 84  0.16666667 0.9100000 0.0010000 0.000
## 85  0.16666667 0.8409091 0.9402274 0.000
## 86  0.10000000 0.8516746 0.0010000 0.000
## 87  0.10000000 0.9523810 0.9402274 0.000
## 88  0.06666667 0.9864253 0.9402274 0.000
## 89  0.13333333 0.9259259 0.0010000 0.000
## 90  0.33333333 0.8750000 0.9402274 0.000
## 91  0.10000000 0.9659091 0.9402274 0.000
## 92  0.10000000 0.9750000 0.0010000 0.000
## 93  0.10000000 0.8888889 0.9402274 0.000
## 94  0.03333333 0.9819005 0.9402274 0.000
## 95  0.03333333 0.9768519 0.9402274 0.000
## 96  0.13333333 0.8352273 0.0010000 0.000
## 97  0.13333333 0.8850000 0.0010000 0.000
## 98  0.13333333 0.9425837 0.9402274 0.000
## 99  0.20000000 0.8101852 0.0010000 0.000
## 100 0.16666667 0.9241071 0.0010000 0.000
```

```
mean(df_log_reg_elnet$MCE)
```

```
## [1] 0.138
```

```
mean(df_log_reg_elnet$AUC)
```

```
## [1] 0.9044936
```

2.8 CART

Next, we fit a classification tree to the data, see Section 2.2 of the paper for details on the method.

```
library("rpart")
```

```
cart_auc <- rep(0, iter)
cart_ce <- rep(0, iter)
set.seed(421)
for(i in 1:iter){
  split_breast= initial_split(data = df, prop = 0.7)
```

```r
train_breast = training(split_breast)
test_breast = testing(split_breast)

tune_spec <-
  decision_tree(
    cost_complexity = tune(),
    tree_depth = tune(),
  ) %>%
  set_engine("rpart", parms = list(split = "information")) %>%
  set_mode("classification")
tune_spec

metrics = metric_set(mn_log_loss)

tree_grid <- grid_regular(cost_complexity(),
                          tree_depth(),
                          levels = 5)

tree_wf <- workflow() %>%
  add_model(tune_spec) %>%
  add_formula(diagnosis_result ~ .)

tv_folds = vfold_cv(train_breast)


tree_res <-
  tree_wf %>%
  tune_grid(
    resamples = tv_folds,
    grid = tree_grid,
    metrics = metrics,
  )

tree_res

h <- tree_res %>%
  collect_metrics()

tree_res %>%  show_best("mn_log_loss")

best_tree <- tree_res %>%
  select_best("mn_log_loss")

best_tree

final_wf <-
  tree_wf %>%
  finalize_workflow(best_tree)

final_fit <-
  final_wf %>%
  last_fit(split_breast, metrics = metric_set(accuracy, roc_auc))
```

```
  estims = final_fit %>%
    collect_metrics()

  cart_auc[i] <- estims$.estimate[2]
  cart_ce[i] <- 1-estims$.estimate[1]
}

df_cart <- data.frame("MCE" = cart_ce, "AUC" = cart_auc)
df_cart
```

```
##             MCE       AUC
## 1   0.23333333 0.7583732
## 2   0.16666667 0.8923445
## 3   0.13333333 0.9166667
## 4   0.23333333 0.7760181
## 5   0.10000000 0.9000000
## 6   0.20000000 0.8054299
## 7   0.16666667 0.7708333
## 8   0.16666667 0.8157895
## 9   0.23333333 0.8253968
## 10  0.13333333 0.9047619
## 11  0.16666667 0.8287037
## 12  0.20000000 0.7655502
## 13  0.20000000 0.8540670
## 14  0.13333333 0.8564815
## 15  0.13333333 0.8733032
## 16  0.10000000 0.9151786
## 17  0.10000000 0.9365079
## 18  0.13333333 0.8750000
## 19  0.23333333 0.7714932
## 20  0.16666667 0.8174603
## 21  0.20000000 0.7840909
## 22  0.10000000 0.8775000
## 23  0.03333333 0.9570136
## 24  0.16666667 0.7942584
## 25  0.20000000 0.8054299
## 26  0.16666667 0.8839286
## 27  0.10000000 0.9000000
## 28  0.26666667 0.7500000
## 29  0.26666667 0.7361111
## 30  0.16666667 0.8110048
## 31  0.30000000 0.6000000
## 32  0.13333333 0.8666667
## 33  0.20000000 0.8000000
## 34  0.23333333 0.7200957
## 35  0.10000000 0.9318182
## 36  0.20000000 0.8200000
## 37  0.20000000 0.8035714
## 38  0.16666667 0.8348416
## 39  0.23333333 0.7638889
## 40  0.13333333 0.9090909
## 41  0.20000000 0.7692308
## 42  0.23333333 0.7484472
## 43  0.20000000 0.8571429
```

```
## 44  0.16666667 0.8333333
## 45  0.16666667 0.8500000
## 46  0.16666667 0.8500000
## 47  0.16666667 0.8438914
## 48  0.13333333 0.8238636
## 49  0.13333333 0.8121693
## 50  0.26666667 0.7142857
## 51  0.23333333 0.7800000
## 52  0.13333333 0.8666667
## 53  0.13333333 0.8250000
## 54  0.13333333 0.7897727
## 55  0.10000000 0.9236111
## 56  0.13333333 0.8666667
## 57  0.23333333 0.7723214
## 58  0.20000000 0.8529412
## 59  0.20000000 0.8311111
## 60  0.16666667 0.8465909
## 61  0.16666667 0.8333333
## 62  0.13333333 0.8666667
## 63  0.20000000 0.7443182
## 64  0.16666667 0.8750000
## 65  0.13333333 0.8947368
## 66  0.10000000 0.8968254
## 67  0.23333333 0.8155556
## 68  0.20000000 0.8229665
## 69  0.30000000 0.6900452
## 70  0.20000000 0.8054299
## 71  0.20000000 0.8194444
## 72  0.10000000 0.8684211
## 73  0.23333333 0.7638889
## 74  0.13333333 0.8947368
## 75  0.16666667 0.8250000
## 76  0.30000000 0.8055556
## 77  0.13333333 0.8730159
## 78  0.20000000 0.7638889
## 79  0.16666667 0.8055556
## 80  0.20000000 0.7443182
## 81  0.16666667 0.8301435
## 82  0.16666667 0.8733032
## 83  0.13333333 0.8095238
## 84  0.36666667 0.6500000
## 85  0.23333333 0.8157895
## 86  0.10000000 0.9537037
## 87  0.20000000 0.8229665
## 88  0.13333333 0.8500000
## 89  0.16666667 0.8348416
## 90  0.13333333 0.8500000
## 91  0.16666667 0.8174603
## 92  0.16666667 0.8958333
## 93  0.16666667 0.8634259
## 94  0.16666667 0.8110048
## 95  0.26666667 0.7104072
## 96  0.03333333 0.9782609
## 97  0.13333333 0.8611111
```

```
## 98  0.16666667 0.8174603
## 99  0.13333333 0.8750000
## 100 0.23333333 0.7579186
```

```
mean(df_cart$MCE)
```

```
## [1] 0.175
```

```
mean(df_cart$AUC)
```

```
## [1] 0.8275257
```

2.9 Random Forest

An example of ensembling is the random forest, which is explained in Section 2.3. By aggregating several trees, the variance of the predictions can be enhanced.

```
library("caret")
library("MLmetrics")
library("randomForestSRC")
```

```
rf_ce <- rep(0, iter)
rf_auc <- rep(0, iter)
num_iters <- 50

for(i in 1:iter){

  train_set = sample(1:length(df[,1]), 0.7 * length(df[,1]))
  test_set = setdiff(1:length(df[,1]), train_set)
    config_ll <- rep(0, num_iters)
  num.trees_val <- sample(250:1500, size = num_iters, replace = T)
  mtry_val <- sample(2:9, size = num_iters, replace = T)
  min.node.size_val <- sample(1:30, size = num_iters, replace = T)
  nodedepth_val <- sample(2:15, size = num_iters, replace = T)
  config_df <- data.frame("num.trees_val" = num.trees_val, "mtry_val" = mtry_val,
                          "min.node.size_val" = min.node.size_val,
                          "nodedepth_val" = nodedepth_val,
                          "config_ll" = config_ll)
  train2 <- createFolds(df[train_set, ]$diagnosis_result, k = 10,
                        list = TRUE, returnTrain = T)
  valid <- lapply(train2, function(x)
    setdiff(1:length(df[train_set,]$diagnosis_result), x))

  for(j in 1:num_iters){
    ll <- rep(0, 10)
    for(k in 1:10){
      modRFSRC <- rfsrc(diagnosis_result ~ ., data = df[train2[[k]],],
                        ntree=config_df$num.trees_val[j],
                        nodesize = config_df$min.node.size_val[j],
                        mtry = config_df$mtry_val[j], splitrule = "entropy",
                        nodedepth = config_df$nodedepth_val[j])
      predictions <- predict(modRFSRC, df[valid[[k]],])
      p <-  predictions$predicted[,"1"]

      true <- as.numeric(df[valid[[k]],]$diagnosis_result == "1")
```

```
      ll[k] <- LogLoss(p, true)
    }
    config_df$config_ll[j] <- mean(ll)
  }

  b <- which.min(config_df$config_ll)
  final_rf <- rfsrc(diagnosis_result ~ ., data = df[train_set,],
                    ntree=config_df$num.trees_val[b],
                    nodesize = config_df$min.node.size_val[b],
                    mtry = config_df$mtry_val[b], splitrule = "entropy",
                    nodedepth = config_df$nodedepth_val[b])
  final_preds <- predict(final_rf, df[test_set,])
  p <-  final_preds$predicted[,"1"]
  true <- as.numeric(df[test_set,]$diagnosis_result == "1")


  final_ce <- sum(round(p) != true)/length(test_set)
  roc_pred <- roc(true, p)
  final_auc <- roc_pred$auc
  rf_auc[i] <- final_auc
  rf_ce[i] <- final_ce
}
df_rf <- data.frame("MCE" = rf_ce, "AUC" = rf_auc)
df_rf
```

```
##           MCE       AUC
## 1   0.26666667 0.8500000
## 2   0.13333333 0.9153439
## 3   0.10000000 0.9772727
## 4   0.13333333 0.7942584
## 5   0.23333333 0.7268519
## 6   0.20000000 0.8250000
## 7   0.20000000 0.9120370
## 8   0.30000000 0.7450000
## 9   0.16666667 0.8371041
## 10  0.16666667 0.8835979
## 11  0.16666667 0.9521531
## 12  0.20000000 0.7731481
## 13  0.20000000 0.8500000
## 14  0.10000000 0.9444444
## 15  0.16666667 0.8416290
## 16  0.16666667 0.9090909
## 17  0.20000000 0.8597285
## 18  0.16666667 0.9095023
## 19  0.23333333 0.8900000
## 20  0.26666667 0.7272727
## 21  0.06666667 0.9700000
## 22  0.10000000 0.9617225
## 23  0.30000000 0.7916667
## 24  0.16666667 0.8616071
## 25  0.03333333 1.0000000
## 26  0.13333333 0.8839286
## 27  0.20000000 0.7142857
## 28  0.26666667 0.8303571
```

```
## 29  0.13333333 0.8750000
## 30  0.16666667 0.8636364
## 31  0.23333333 0.9350000
## 32  0.10000000 0.8888889
## 33  0.06666667 0.9282297
## 34  0.23333333 0.8794643
## 35  0.23333333 0.7037037
## 36  0.23333333 0.8258929
## 37  0.16666667 0.9617225
## 38  0.26666667 0.6877828
## 39  0.23333333 0.8086124
## 40  0.10000000 0.8899522
## 41  0.13333333 0.8687783
## 42  0.30000000 0.7777778
## 43  0.16666667 0.7460317
## 44  0.13333333 0.8235294
## 45  0.23333333 0.8660287
## 46  0.33333333 0.7737557
## 47  0.20000000 0.7942584
## 48  0.13333333 0.8868778
## 49  0.26666667 0.7556561
## 50  0.30000000 0.7300000
## 51  0.23333333 0.8201058
## 52  0.10000000 0.8914027
## 53  0.13333333 0.7888199
## 54  0.16666667 0.9090909
## 55  0.10000000 0.8708134
## 56  0.23333333 0.6842105
## 57  0.26666667 0.7901786
## 58  0.23333333 0.7129187
## 59  0.16666667 0.8730159
## 60  0.30000000 0.7420814
## 61  0.13333333 0.8687783
## 62  0.26666667 0.7873303
## 63  0.40000000 0.7767857
## 64  0.23333333 0.8044444
## 65  0.23333333 0.9043062
## 66  0.26666667 0.7638889
## 67  0.16666667 0.8200000
## 68  0.23333333 0.8235294
## 69  0.33333333 0.8687783
## 70  0.33333333 0.8928571
## 71  0.10000000 0.9722222
## 72  0.23333333 0.8750000
## 73  0.33333333 0.7950000
## 74  0.16666667 0.8750000
## 75  0.20000000 0.9107143
## 76  0.23333333 0.8009050
## 77  0.20000000 0.8250000
## 78  0.26666667 0.9212963
## 79  0.30000000 0.8054299
## 80  0.23333333 0.7018634
## 81  0.16666667 0.8755981
## 82  0.23333333 0.9351852
```

```
## 83  0.23333333 0.8373206
## 84  0.16666667 0.9234450
## 85  0.23333333 0.8287037
## 86  0.23333333 0.8280543
## 87  0.23333333 0.7619048
## 88  0.16666667 0.8850000
## 89  0.13333333 0.8095238
## 90  0.20000000 0.9186603
## 91  0.13333333 0.8633540
## 92  0.30000000 0.7650000
## 93  0.20000000 0.8666667
## 94  0.26666667 0.7870370
## 95  0.26666667 0.7894737
## 96  0.10000000 0.9553571
## 97  0.30000000 0.8280543
## 98  0.20000000 0.8099548
## 99  0.26666667 0.9111111
## 100 0.13333333 0.9027778
```

```
mean(df_rf$MCE)
```

```
## [1] 0.2036667
```

```
mean(df_rf$AUC)
```

```
## [1] 0.8436553
```

2.10 Subset Selection

As a final comparator, we investigate subset selection. This was also proposed by Hastie et al. (2001) in their analysis of a prostate cancer dataset, see Section 4.1 of the main paper.

```
f <- function(){
  ss_auc <- rep(0,iter)
  ss_ce <- rep(0,iter)
  for(m in 1:iter){
    train_set = sample(1:length(df[,1]), 0.7 * length(df[,1]))
    test_set = setdiff(1:length(df[,1]), train_set)

    vars = names(df)
    vars = vars[-1]

    d_train = sample(train_set, 9/10 * length(train_set))
    d_valid = setdiff(train_set, d_train)
    model = NULL
    bestmod_list <- list()
    bestmod_list[[1]] <- glm(diagnosis_result ~ 1, df[d_train,],
                             family =binomial(link = "logit"))

    bilde_alle_modelle <- function(vars, data, model, bestmod_list){
      for(i in 1:length(vars)){
        xx = combn(vars,i)
        if(is.null(dim(xx))){
          fla = paste("diagnosis_result ~", paste(xx, collapse="+"))
          model[[length(model)+1]]=glm(as.formula(fla),data=data,
                                       family = binomial(link = "logit"))
```

```
    } else {
      for(j in 1:dim(xx)[2]){
        fla = paste("diagnosis_result ~", paste(xx[1:dim(xx)[1],j],
                                               collapse="+"))
        model[[length(model)+1]]=glm(as.formula(fla),data=data,
                                    family = binomial(link = "logit"))
        if(j != 1){
          if(glm(as.formula(fla),data=data,
  family = binomial(link = "logit"))$deviance < bestmod_list[[i]]$deviance){
            bestmod_list[[i+1]] <- glm(as.formula(fla),data=data,
                                       family = binomial(link = "logit"))
          }
        } else{
          bestmod_list[[i+1]] <- glm(as.formula(fla),data=data,
                                    family = binomial(link = "logit"))
        }
      }
    }
  }
  return(list(model, bestmod_list))
}
output <- bilde_alle_modelle(vars = vars, data = df[d_train,], model = model,
                             bestmod_list = bestmod_list)
bestmod_list <- output[[2]]
model <- output[[1]]

model[[2^8]] = glm(diagnosis_result ~ 1, df[d_train,],
                   family =binomial(link = "logit"))
log_losses <- rep(0,9)
for(k in 1:length(bestmod_list)){
  predictions <- predict.glm(bestmod_list[[k]], newdata = df[d_valid,],
                             type = "response")
  log_losses[k] <- LogLoss(predictions,
                           as.numeric(df[d_valid,]$diagnosis_result))
}


bestmodel <- bestmod_list[[which(min(log_losses) == log_losses)]]
names(bestmodel$coefficients)
if(length(names(bestmodel$coefficients)) != 1){
  h <- as.character(bestmodel$formula)
  anzahl_variablen <- ceiling(length(strsplit(h[3], " ")[[1]])/2)
  xx = combn(names(df)[-1],anzahl_variablen)
  for(j in 1:dim(xx)[2]){
    fla = paste("diagnosis_result ~",
                paste(xx[1:dim(xx)[1],j], collapse="+"))
    if(j != 1){
      if(glm(as.formula(fla),data=df[train_set,],
         family = binomial(link = "logit"))$deviance < bestmodel$deviance){
        bestmodel <- glm(as.formula(fla),data=df[train_set,],
                         family = binomial(link = "logit"))
      }
    } else{
```

```r
          bestmodel <- glm(as.formula(fla),data=df[train_set,],
                           family = binomial(link = "logit"))
        }
      }

    }
    if(length(names(bestmodel$coefficients)) == 1){
      bestmodel <- glm(diagnosis_result ~ 1, df[train_set,],
                       family =binomial(link = "logit"))
    } else{
      bestmodel <- glm(bestmodel$formula,
                       df[train_set,], family = binomial(link = "logit"))
    }


    final_predictions <- predict.glm(bestmodel, df[test_set,], type = "response")
    final_ce <- sum(round(final_predictions)!=df[test_set,]$diagnosis_result)/length(test_set)
    roc_pred <- roc(df[test_set,]$diagnosis_result, final_predictions, quiet = T)
    final_auc <- roc_pred$auc
    ss_auc[m] <- final_auc
    ss_ce[m] <- final_ce
  }
  df_ss <- data.frame("MCE" = ss_ce, "AUC" = ss_auc)
  return(df_ss)
}
```

```r
df_ss <- f()
df_ss
```

```
##             MCE       AUC
## 1   0.30000000 0.8500000
## 2   0.16666667 0.8518519
## 3   0.26666667 0.7963801
## 4   0.26666667 0.7963801
## 5   0.46666667 0.5000000
## 6   0.16666667 0.9600000
## 7   0.20000000 0.8600000
## 8   0.20000000 0.8711111
## 9   0.10000000 0.8941799
## 10  0.13333333 0.8947368
## 11  0.23333333 0.9185520
## 12  0.46666667 0.5000000
## 13  0.23333333 0.9090909
## 14  0.26666667 0.8660287
## 15  0.20000000 0.9000000
## 16  0.16666667 0.9629630
## 17  0.03333333 0.9700000
## 18  0.16666667 0.9300000
## 19  0.23333333 0.8705357
## 20  0.23333333 0.7500000
## 21  0.20000000 0.7824074
## 22  0.23333333 0.8708134
## 23  0.20000000 0.9234450
## 24  0.10000000 0.9880383
```

```
## 25  0.16666667 0.9100000
## 26  0.16666667 0.9488636
## 27  0.13333333 0.9150000
## 28  0.20000000 0.8612440
## 29  0.20000000 0.9488889
## 30  0.36666667 0.7566138
## 31  0.23333333 0.8144796
## 32  0.23333333 0.8928571
## 33  0.16666667 0.8518519
## 34  0.20000000 0.8229665
## 35  0.10000000 0.9365079
## 36  0.13333333 0.9250000
## 37  0.30000000 0.7901786
## 38  0.16666667 0.8133971
## 39  0.10000000 0.9186603
## 40  0.16666667 0.9140271
## 41  0.23333333 0.8552036
## 42  0.16666667 0.9244444
## 43  0.26666667 0.7936508
## 44  0.20000000 0.8277512
## 45  0.13333333 0.8687783
## 46  0.26666667 0.7799043
## 47  0.06666667 0.9185520
## 48  0.26666667 0.8947368
## 49  0.20000000 0.8506787
## 50  0.16666667 0.9234450
## 51  0.46666667 0.5000000
## 52  0.20000000 0.9377990
## 53  0.16666667 0.9300000
## 54  0.16666667 0.8571429
## 55  0.26666667 0.6722488
## 56  0.16666667 0.9212963
## 57  0.30000000 0.7936508
## 58  0.16666667 0.8506787
## 59  0.40000000 0.5000000
## 60  0.10000000 0.8350000
## 61  0.36666667 0.5000000
## 62  0.16666667 0.8806818
## 63  0.26666667 0.8235294
## 64  0.23333333 0.8444444
## 65  0.23333333 0.8125000
## 66  0.23333333 0.8516746
## 67  0.26666667 0.8181818
## 68  0.36666667 0.5000000
## 69  0.13333333 0.9062500
## 70  0.20000000 0.8564593
## 71  0.16666667 0.8973214
## 72  0.26666667 0.8348214
## 73  0.06666667 0.9120370
## 74  0.16666667 0.8950000
## 75  0.16666667 0.9200000
## 76  0.20000000 0.9120370
## 77  0.13333333 0.9768519
## 78  0.13333333 0.9147727
```

```
## 79  0.13333333 0.9629630
## 80  0.23333333 0.8650000
## 81  0.20000000 0.9192547
## 82  0.16666667 0.9400000
## 83  0.20000000 0.8850000
## 84  0.20000000 0.8794643
## 85  0.13333333 0.9444444
## 86  0.13333333 0.8935185
## 87  0.10000000 0.9550000
## 88  0.10000000 0.9400000
## 89  0.06666667 0.9808612
## 90  0.13333333 0.8657407
## 91  0.20000000 0.8823529
## 92  0.13333333 0.8835979
## 93  0.13333333 0.8465608
## 94  0.20000000 0.8516746
## 95  0.23333333 0.8687783
## 96  0.23333333 0.9351852
## 97  0.30000000 0.7724868
## 98  0.23333333 0.6825397
## 99  0.13333333 0.9683258
## 100 0.20000000 0.9241071
```

```
mean(df_ss$MCE)
```

```
## [1] 0.202
```

```
mean(df_ss$AUC)
```

```
## [1] 0.8557543
```

2.11 Summary

Finally, we summarize the results of the different approaches with respect to AUC and MCE here:

```
AUC <- data.frame("log_reg" = mean(df_log_reg$AUC),
                  "ridge" = mean(df_log_reg_ridge$AUC),
                  "lasso" = mean(df_log_reg_lasso$AUC),
                  "elnet" = mean(df_log_reg_elnet$AUC),
                  "ctree" = mean(df_cart$AUC), "forest" = mean(df_rf$AUC),
                  "subset_selection" = mean(df_ss$AUC))
AUC
```

```
##     log_reg     ridge     lasso     elnet     ctree    forest subset_selection
## 1 0.8836972 0.9044936 0.8957995 0.9044936 0.8275257 0.8436553        0.8557543
```

```
MCE <- data.frame("log_reg" = mean(df_log_reg$MCE),
                  "ridge" = mean(df_log_reg_ridge$MCE),
                  "lasso" = mean(df_log_reg_lasso$MCE),
                  "elnet" = mean(df_log_reg_elnet$MCE),
                  "ctree" = mean(df_cart$MCE), "forest" = mean(df_rf$MCE),
                  "subset_selection" = mean(df_ss$MCE))
MCE
```

```
##     log_reg ridge lasso elnet ctree    forest subset_selection
## 1 0.1833333 0.138 0.261 0.138 0.175 0.2036667            0.202
```

```r
AUC_with_interactions <- data.frame("log_reg" = mean(df_log_reg$AUC),
                    "ridge" = mean(df_log_reg_ridge$AUC),
                    "lasso" = mean(df_log_reg_lasso$AUC),
                    "elnet" = mean(df_log_reg_elnet$AUC),
                    "full_two_way" = mean(logreg_full_twoway_auc),
                    "compactness" = mean(logreg_compactness_auc),
                    "ctree" = mean(df_cart$AUC), "forest" = mean(df_rf$AUC),
                    "subset_selection" = mean(df_ss$AUC))

MCE_with_interactions <- data.frame("log_reg" = mean(df_log_reg$MCE),
                    "ridge" = mean(df_log_reg_ridge$MCE),
                    "lasso" = mean(df_log_reg_lasso$MCE),
                    "elnet" = mean(df_log_reg_elnet$MCE),
                    "full_two_way" = mean(logreg_full_twoway_mce),
                    "compactness" = mean(logreg_compactness_mce),
                    "ctree" = mean(df_cart$MCE), "forest" = mean(df_rf$MCE),
                    "subset_selection" = mean(df_ss$MCE))

AUC_with_interactions
```

```
##     log_reg     ridge     lasso     elnet full_two_way compactness     ctree
## 1 0.8836972 0.9044936 0.8957995 0.9044936    0.6643755   0.7919776 0.8275257
##      forest subset_selection
## 1 0.8436553        0.8557543
```

```r
MCE_with_interactions
```

```
##     log_reg ridge lasso elnet full_two_way compactness ctree    forest
## 1 0.1833333 0.138 0.261 0.138        0.348   0.2386667 0.175 0.2036667
##   subset_selection
## 1            0.202
```

	MCE	AUC
Log. Regression	0.18	0.88
Ridge	0.14	0.90
LASSO	0.26	0.90
Elastic Net	0.14	0.90
Log. Regression with all two-way interactions	0.35	0.66
Log. Regression with all two-way interactions involving compactness	0.24	0.79
Classification Tree	0.17	0.83
Random Forest	0.20	0.84
Subset Selection	0.20	0.86

Table 2: Mean results for MCE and AUC of the methods used in this example.

3 Number of Downloads of R packages

To give an idea of how frequently the methods described in the main paper are applied, we extracted the number of CRAN downloads for each package mentioned in the paper. The results are displayed in the following table:

Package	2017	2018	2019	2020
BMA	37536	44432	39834	79725
BMS	11037	10372	13980	22163
deepnet	14665	22953	31425	40513
gbm	186378	223004	321120	373363
glmnet	445589	500287	737619	842774
keras	23248	141261	243913	367732
mboost	53657	58106	59723	94969
mlr3	0	0	5013	121672
MuMIn	68702	94512	138151	172693
neuralnet	147558	158533	213081	224475
party	321007	331180	503582	699529
penalized	37658	45540	48669	68328
ranger	86022	142972	396096	1016793
xgboost	160476	260435	590513	950533

Table 3: Number of downloads of the respective R-packages for the years 2017 - 2020.
